# TRIM46 activates AKT/HK2 signaling by modifying PHLPP2 ubiquitylation to promote glycolysis and chemoresistance of lung cancer cells

**DOI:** 10.1038/s41419-022-04727-7

**Published:** 2022-03-30

**Authors:** Jicheng Tantai, Xufeng Pan, Yong Chen, Yuzhou Shen, Chunyu Ji

**Affiliations:** Department of Thoracic Surgery, Shanghai Chest Hospital, Shanghai Jiao Tong University, Shanghai, 200030 China

**Keywords:** Cancer, Biomarkers

## Abstract

The incidence of lung cancer is increasing worldwide. Although great progress in lung cancer treatment has been made, the clinical outcome is still unsatisfactory. Tripartite motif (TRIM)-containing proteins has been shown to be closely related to tumor progression. However, the function of TRIM46 in lung cancer is largely unknown. Here, TRIM46 amplification was found in lung adenocarcinoma (LUAD) tissues and TRIM46 amplification was significantly associated with a poor survival rate. Overexpression of wild type TRIM46 increased the proliferation of LUAD cells and glycolysis, promoted xenografts growth, and enhanced cisplatin (DDP) resistance of LUAD cells via increased ubiquitination of pleckstrin homology domain leucine-rich repeat protein phosphatase 2 (PHLPP2) and upregulation of p-AKT. In contrast, overexpression of RING-mutant TRIM46 did not show any effects, suggesting the function of TRIM46 was dependent on the E3 ligase activity. Furthermore, we found that TRIM46 promoted LUAD cell proliferation and DDP resistance by enhancing glycolysis. PHLPP2 overexpression reversed the effects of TRIM46 overexpression. Amplification of TRIM46 also promoted LUAD growth and enhanced its DDP resistance in a patient-derived xenograft (PDX) model. In conclusion, our data highlight the importance of TRIM46/PHLPP2/AKT signaling in lung cancer and provide new insights into therapeutic strategies for lung cancer.

## Introduction

Lung cancer is the most frequent malignant neoplasm in most countries [1], with common symptoms of cough, dyspnea, or hemoptysis [2]. It has been estimated that 135,720 people will die from lung cancer & bronchus cancer in 2020 in USA [3]. Risk factors for lung cancer include genetic risk factors, behavioral and environmental factors such as tobacco smoking, diet, and alcohol, all of which play a part in tumor development [4]. Lung adenocarcinoma (LUAD), a very aggressive subtype of lung cancer, possesses strong heterogeneities in both tumor biology and clinical characteristics [5, 6]. Although great progress in lung cancer treatment has been made, the clinical outcome is still unsatisfactory.

Glycolysis is the central pathway for the glucose catabolism in which glucose is converted into pyruvate and energy in the form of adenosine triphosphate (ATP) is produced [7]. Studies indicated that tumor cells have different metabolic pathways than normal cells. For example, it has been shown that there is a metabolic switch from oxidative phosphorylation (OXPHOS) to glycolysis in cancer cells and cancer cells can derive their energy from glycolysis that is glucose is converted to lactate for energy followed by lactate fermentation, even when oxygen is available [8]. Lee et al. has reported that glycolytic enzymes were upregulated in precancerous cirrhotic livers and contributed to the development of hepatocellular carcinoma (HCC) [9]. A study by Hensley et al. has shown that glycolysis was significantly elevated in non-small cell lung cancer (NSCLC) [10]. Clinically, elevated GLUT1-mediated glycolysis in lung squamous cell carcinoma strongly correlates with poor prognosis [11]. Furthermore, glycolysis has been implicated in chemo-resistance of cancer cells. For instance, a study has reported that the glycolysis activity of drug-resistant HCC cell lines is significantly upregulated [12]. Inhibition of GLUT-1/Akt has been shown to promote chemosensitivity [13]. Previous studies also have found that downregulating Hexokinases II (HK2)-mediated glycolysis by Deguelin or downregulating ATK-mediated glycolysis by miR-128 suppresses the progression of lung cancer [14, 15], suggesting that glycolysis suppression might be a strategy for drug-resistant LUAD treatment.

Tripartite motif (TRIM)-containing proteins are a family of approximately 70 proteins that have an N-terminal RING finger, one or two B-boxes, and a coiled-coil (CC) domain [16]. RING finger structure is closely related to E3 ubiquitin ligases activity [17]. Although not all TRIM proteins have E3 activity, TRIM proteins have attracted attentions in recent years from E3 research field [17]. Studies suggested that TRIM proteins are closely related to tumor progression. For example, TRIM24 accelerates cell proliferation, and promotes the progression of prostate cancer [18]. Han et al. has shown that TRIM15 was significantly upregulated in NSCLC tissues and TRIM15 could act as a therapeutic target for lung cancer treatments [17]. A study by Jiang et al. has demonstrated that TRIM46 promotes viability and inhibits apoptosis of osteosarcoma cells by activating NF-κB signaling through ubiquitination of PPARα [19]. TRIM46 has also been shown to control neuronal polarity and axon specification by driving the formation of parallel microtubule arrays [20]. Nevertheless, the function of TRIM46 in lung cancer especially LUAD is largely unknown.

Copy number variation (CNV) is defined as an increasing or decreasing number of DNA segments (>1 kb) [21]. Increasing evidence suggests CNVs of certain genes are implicated in various types of cancers [21]. For instance, it has been shown that neurofascin is dramatically amplified in NSCLC patients and functions as a regulator of NSCLC cell motility [22]. Zhang et al. has reported that amplification of epidermal growth factor receptor was associated with higher objective response rates in NSCLC patients receiving tyrosine kinase inhibitor treatment [23]. However, whether TRIM46 is amplified and the role of TRIM46 amplification in lung cancer are largely unknown.

The aims of this study were to elucidate the function of TRIM46 in lung cancer and chemo-resistance of lung cancer cells, and to decipher what molecular pathways were necessary for such effects and the underlying mechanisms.

## Material and methods

### Bioinformatics analysis

LUAD datasets were downloaded from Cancer Genome Atlas (TCGA) (https://tcga-data.nci.nih.gov/tcga/). DNA copy-number, gene expression, and gene set enrichment analysis (GSEA) were applied [24].

### Study subjects

The protocol conforms to ethical guidelines of Declaration of Helsinki, and has the approval from Institutional Ethical Review Committee of Shanghai Chest Hospital, Shanghai Jiao Tong University (Shanghai, China, approval number: KS(Y)21210). LUAD patients had surgeries from January 2007 to December 2008 at Shanghai Chest Hospital, Shanghai Jiao Tong University (Shanghai, China) were included after written informed consents were given. Cohort 1 included 72 patients with fresh tissues, cohort 2 had 80 formalin-fixed, paraffin-embedded tissues.

### CNV analysis

Genomic DNA was isolated form 72 LUAD samples (cohort 1) using a Magnetic Tissue DNA Kit (Beyotime, Suzhou). TRIM46 copy number was measured using a QX200 Droplet Digital PCR cycler (Bio-Rad, Shanghai) (TRIM46: primers, 5′-ACGGCGAATACAGTGAAGATG-3′ and 5′-TAGGAGGCTGGGTCTACGG-3′, target probe: 5′-FAMCTGGCTATCAGCAAGGACCAGCGAGBHQ1-3′).

### RNA isolation and quantitative RT-PCR

RNAs were isolated using Trizol (Invitrogen). TRIM46 expression was measured by qRT-PCR using the SYBR Green mix and an ABI7300 cycler (ABI), using GAPDH as an internal control. The primers are as follows: TRIM46, 5′-CGCCTGGTATGTCAACTC-3′ and 5′-CTCCTGCTGGCATTCTTC-3′; PHLPP2, 5′-GTTAGAAAGATGGGAACAAGAG-3′ and 5′-AAGTAGGTTGGGAAACGAATAG-3′; GAPDH, 5′-AATCCCATCACCATCTTC-3′ and 5′-AGGCTGTTGTCATACTTC-3′. Cycling parameters were, 95 °C, 10 min, 40 cycles of (95 °C, 15 s, 60 °C, 45 s). Gene fold-changes were calculated using 2^−△△CT^.

### Immunohistochemistry (IHC) analysis

Tissue sections were deparaffinized, antigen-retrieved, hydrogen peroxide (0.3%) treated, blocked, incubated with antibody against TRIM46 (bs-16740R, Bioss), PHLPP2 (ab227673, Abcam) or p-AKT (S473)(ab8805, Abcam) for 12 h at 4 °C, then incubated with HRP-conjugated 2nd antibody for 1 h, stained with DAB and hematoxylin. Images were taken with microscope at 200 magnification (Nikon, Japan). Immunoreactivity was scored using the H-score system by two investigators based on the percentage of positively stained cells (graded on a scale of 0–4: 0, <5%; 1, 5–25%; 2, 25–50%; 3, 50–75%; 4, >75%). A staining index ≥ 3 was considered as overexpression.

### Cell culture

HEK293T, human bronchial epithelial cell line (16HBE), and human LUAD cell lines, H358, SKMES1, H1299, HCC827, H292, H1975, and A549 were obtained from Shanghai Cell Bank and authenticated by STR before shipping. Mycoplasma contamination was tested if concerned. Lentiviral-based CRISPR gene editing system (LentiCRISPR) was used to establish PHLPP2 knockout H358 cells as previously described [25]. Cells were maintained in DMEM with 10% FBS.

### Plasmid construction and virus production

Short hairpin RNA (shRNA) oligos targeting TRIM46 (shTRIM46-1, 5′-GGTGAGGATATGCAGACCT-3′; shTRIM46-2, 5′-GATATGCAGACCTTCACTT-3′) were ligated into pLKO.1 (Addgene, Watertown, MA). Wild type human TRIM46 (TRIM46 WT), RING mutant human TRIM46 (TRIM46 WT) [20], and PHLPP2 was synthesized by Genwiz (Suzhou, China) and inserted into pLVX-puro (Addgene, Watertown, MA). Virus of shTRIM46, control shRNA (shNC), pLVX-TRIM46 (oeTRIM46), pLVX-PHLPP2 (oePHLPP2), or pLVX-puro (Vector) was produced in 293T cells.

### Western blots

Proteins were extracted with RIPA buffer, separated by SDS-PAGE, transferred to PVDF membranes, blocked, incubated with primary antibodies (Table S1), washed, and incubated with secondary antibody. Signal was detected using enhanced chemiluminescence system (ECL) (Millipore).

### Ethynyl-2-deoxyuridine (EdU) assay

Cell proliferation was determined by EdU staining using a BeyoClick™ EdU Cell Proliferation Kit (Beyotime, Shanghai). Briefly, cells were incubated with 20 μM EdU for 2 h. After washing with PBS, cells were fixed in 4% polyformaldehyde, permeabilized with 0.3% Triton X-100, and treated with Click Additive Solution for 30 min. After incubating with DAPI solution, cells were visualized under fluorescence microscope at 400 magnification (Nikon, Japan).

### Immunofluorescence staining

Cells on coverslips were fixed, blocked, incubated with mouse anti-TRIM46 (Abcam, ab169044) or rabbit anti-PHLPP2 (Abcam, ab71973) antibodies overnight at 4 °C, washed, and then incubated with the Alexa Fluor 555-labeled or Alexa Fluor 488-labeled second antibody. DAPI was used to stain nuclei. The cells were observed the fluorescence intensity under the fluorescence microscope at 1000 magnification (Nikon, Japan).

### Animal experiments

Animal experiments had the approval from Animal Care Committee of Shanghai Chest Hospital, Shanghai Jiao Tong University (Shanghai, China, approval number: KS(Y)21210). Male nude mice (12-week) (SLRC, Shanghai) were subcutaneously injected with control or TRIM46-silencing H358 cells, or A549 cells stably expressing TRIM46 (TRIM46 WT), RING-mutant, or Vector (5 × 10^6^ cells per mouse). Tumor growth was tracked every 3 days: volume = 1/2 × (length) × (width)^2^. Mice were euthanized 33 days after inoculation, tumor were collected, weighed, and used for immunoblotting and TUNEL staining (Roche, Indianapolis) according to the manufacturer’s protocol. Apoptotic cells were detected with a microscope at 400 magnification (Nikon, Japan)

Fresh tumor tissues were minced (3 mm^3^) and implanted subcutaneously to nude mice to generate patient-derived xenograft (PDX) model. Mice were randomly separated into three groups (*n* = 5) when tumors reached 100 mm^3^. Cisplatin (DDP, 5 mg/kg/day) or vehicle was given to mice every three days. Tumors were collected at 21 days.

### Measurement of cellular respiration and glycolytic activity

Extracellular acidification rate (ECAR) and oxygen consumption rate (OCR) were measured with a Seahorse XF96 analyzer (North Billerica, MA). Cells were inoculated 24 h before. For examining ECAR, the glyco-stress test kit (Seahorse Bioscience) and a mito-stress kit were used for analysis of ECAR and OCR.

### Immunoprecipitation (IP) assays

Proteins were incubated with anti-TRIM46, anti-PHLPP2 antibody or control IgG for 1 h at 4 °C, then incubated with protein A/G-beads for 3 h at 4 °C. Beads were washed three times and proteins were analyzed by immunoblots.

### Statistical analysis

The statistical significance of mean values was determined by an unpaired two-tailed Student’s *t*-test. The comparison of statistical significance among three or more groups was determined by one-way analysis of variance (ANOVA). Graphpad Prism 8.4.2 (San Diego, CA, USA) was used for statistical analysis. A value of *p* < 0.05 was considered to be of statistical significance.

## Results

### TRIM46 amplification in LUAD was associated with overall survival

**Fig. 1:**
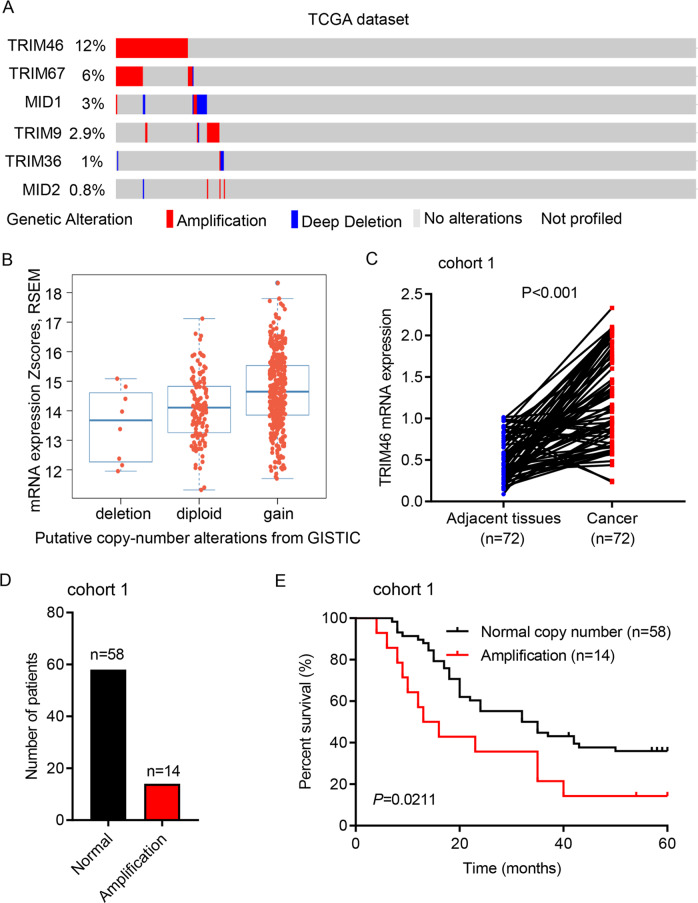
TRIM46 amplification in LUAD was associated with overall survival. **A** CNV analysis of 6 TRIM proteins in TCGA cohort (*n* = 230). **B** TRIM46 amplification was associated with higher TRIM46 mRNA level. **C** Statistical analysis of TRIM46 mRNA expression in LUAD and paired adjacent normal tissues of Shanghai Chest Hospital cohort 1 (*n* = 72, nonparametric Mann–Whitney test). **D** Analysis of TRIM46 copy number by real-time PCR in Shanghai Chest Hospital cohort 1 (*n* = 72). **E** Survival analysis was performed between patients with TRIM46 amplification or normal copy number in Shanghai Chest Hospital cohort 1 (log-rank test).

To evaluate the function of TRIM46 in LUAD, microarrays of 230 LUAD patients from TCGA database were analyzed for 6 TRIM family members. CNV analysis revealed that 6 TRIM family members showed copy-number amplification in patients with LUAD from TCGA dataset, with TRIM46 showed the highest amplification (Fig. 1A). So, TRIM46 was chosen for further study. TRIM46 amplification correlated with a higher TRIM46 mRNA level (Fig. 1B). Clinical samples from Cohort 1 were then used to analyze TRIM46 amplification. Quantitative RT-PCR results suggested that TRIM46 mRNA levels were significantly upregulated in LUAD specimen compared to that of normal tissue (Fig. 1C). Fourteen out of 72 LUAD patients showed TRIM46 amplification (Fig. 1D). The Kaplan–Meier analyses showed that amplification of TRIM46 correlated to poor survival rates (Fig. 1E). These findings suggest that TRIM46 amplification was associated with overall survival of LUAD patients.

### The clinical relevance of TRIM46 in LUAD

**Fig. 2:**
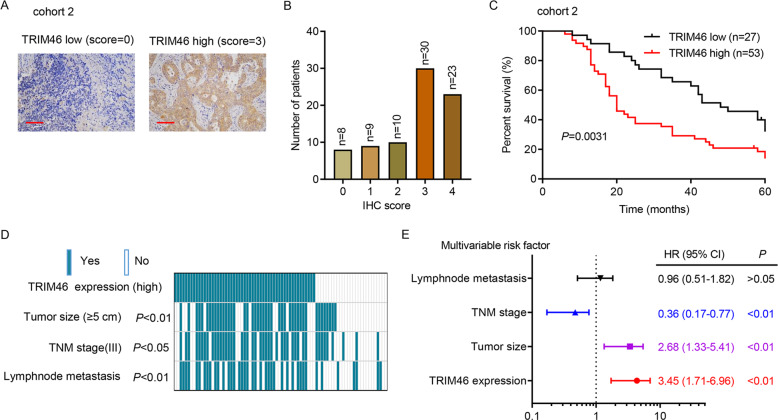
The clinical relevance of TRIM46 in LUAD. **A** Immunohistochemical staining of TRIM46 in Shanghai Chest Hospital cohort 2 (scale bar: 100 μm). **B** Immunohistochemistry score of TRIM46 in Shanghai Chest Hospital cohort 2. **C** Survival analysis was performed between patients with TRIM46 high- and low-expression in Shanghai Chest Hospital cohort 2 (log-rank test). **D** Comparing tumor size, TNM stage, and lymphnode metastasis between TRIM46 high- and low expressing tumors in the Shanghai Chest Hospital cohort 2. The heat map illustrates the association of different clinicopathologic features with TRIM46 high- and low-expression (*n* = 80). **E** Multivariate regression analysis was applied (all the bars correspond to 95% confidence intervals).

To further analyze the clinical relevance of TRIM46 in LUAD, we next analyzed TRIM46 expression in cohort 2. Immunohistochemical staining results showed that 53 patients had a higher level of TRIM46 expression, while 27 patients showed a lower level of TRIM46 expression (Fig. 2A, B). Higher TRIM46 levels correlated to poorer survival rates (Fig. 2C). Comparing tumor size, TNM stage, and lymphnode metastasis between TRIM46 high- and low expressing tumors, we found that TRIM46 expression positively correlated to sizes of tumor, TMN stages, and metastasis (Fig. 2D). Data also indicated that TRIM46 was an independent predictor of LUAD aggressiveness and tumor sizes with significant hazard ratio (HR) (Fig. 2E). Together, TRIM46 is increased and correlated to clinicopathologic characters and poor survival rates in LUAD.

### E3 ligase activity of wild type TRIM46 accounted for increased proliferation of LUAD cells, promoted xenografts growth, and enhanced DDP resistance of LUAD cells

**Fig. 3:**
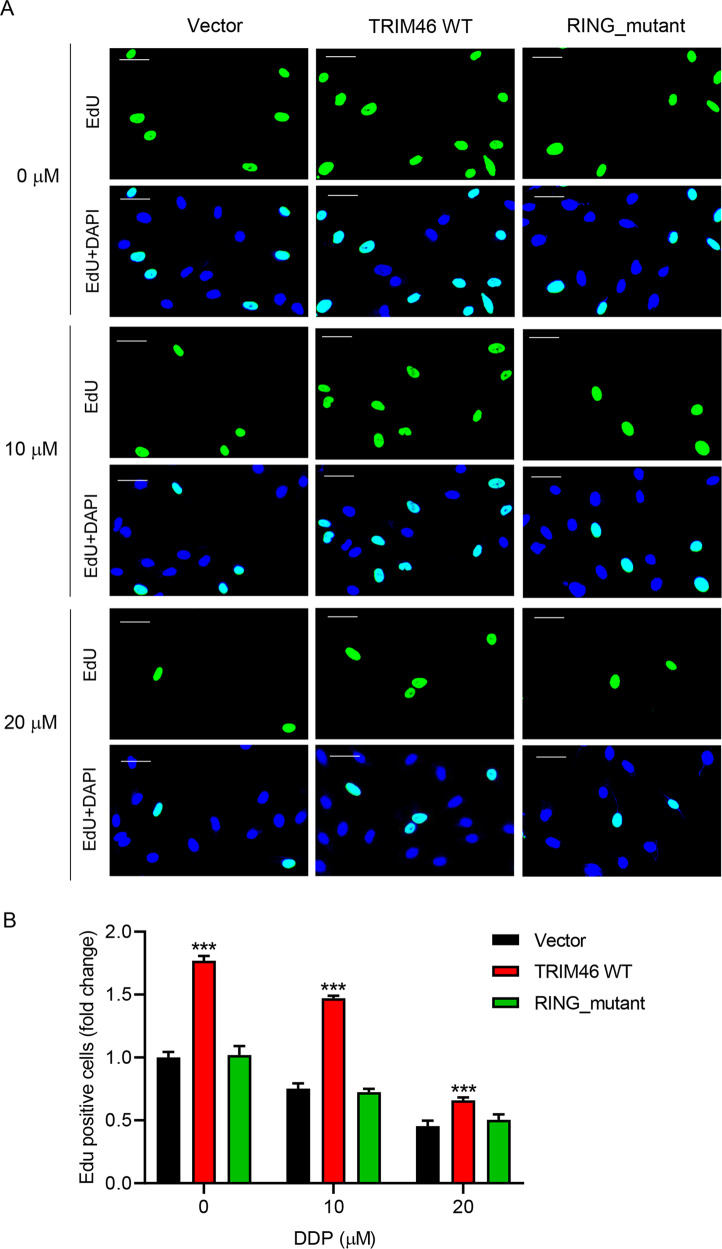
TRIM46 overexpression increased cell proliferation and DDP resistance of LUAD cells. A549 cells were transfected with WT-TRIM46, RING-mutant TRIM46, or Vector and treated by DDP (0, 10, or 20 μM). **A** A549 cell proliferation measured by EdU staining (scale bar: 50 μm). **B** Statistical analysis of EdU positive A549 cells. Results were presented as the mean ± standard error (*n* = 3). Independent experiments were repeated three times. ***P < 0.001 vs Vector.

To further investigate TRIM46 function, WT- or RING-mutant TRIM46 was overexpressed in A549 cells (Fig. S1A, B). Overexpression of WT-TRIM46 significantly increased cell proliferation compared to vector control (Fig. 3A, B). In contrast, overexpression of RING-mutant TRIM46 did not affect A549 cell proliferation. Consequently, overexpression of WT-TRIM46 significantly increased tumor growth and tumor sizes (Fig. S2A, B). TUNEL staining results suggested that overexpression of WT-TRIM46 suppressed apoptosis of A549 cells (Fig. S2C). However, overexpression of RING-mutant TRIM46 did not show any effect on tumor growth, tumor sizes, and cell apoptosis (Fig. S2A–S2C). Overexpression of WT-TRIM46 also decreased DDP-induced suppression of A549 cell proliferation (Fig. 3A, B). However, overexpression of RING-mutant TRIM46 did not show any effect on DDP-induced suppression of A549 cell proliferation (Fig. 3A, B), suggesting that E3 ligase activity played a key role in exerting the function of TRIM46. Silencing of TRIM46 significantly inhibited the growth of H358 and H1299 cells in vitro (Fig. S3A–C) and tumor growth in vivo (Fig. S3D, E). These findings suggest that TRIM46 overexpression increased the proliferation of LUAD cells, promoted xenografts growth, and enhanced DDP resistance of LUAD cells.

### TRIM46 promoted LUAD cell proliferation and DDP resistance by enhancing glycolysis

**Fig. 4:**
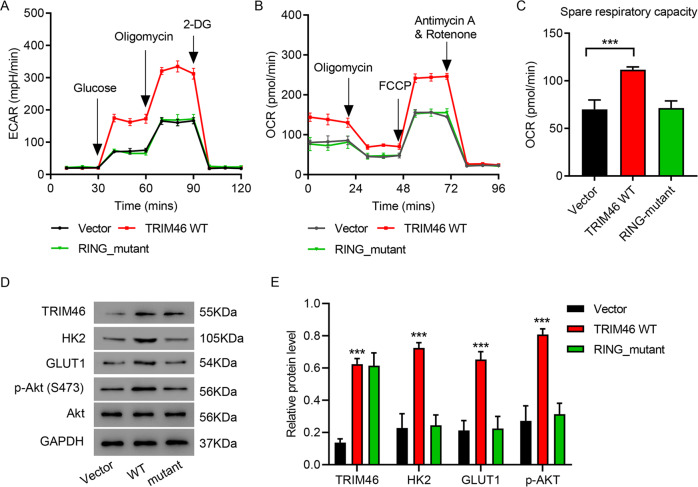
TRIM46 promoted LUAD cell glycolysis. **A** ECAR, **B** OCR, **C** spare respiratory capacity and **D**, **E** expression of TRIM46, AKT, p-AKT, HK2, and GLUT1 of A549 cells transfected with WT-TRIM46, RING-mutant TRIM46, or Vector. Results were presented as the mean ± standard error (*n* = 3). Independent experiments were repeated three times. ***P < 0.001.

Overexpression of WT-TRIM46 also significantly increased ECAR and OCR compared to that of vector controls (Fig. 4A–C). Overexpression of RING-mutant TRIM46 did not affect ECAR or OCR. TRIM46 knockdown inhibited ECAR and OCR in H358 and H1299 cells (Fig. S4A–S4F). To investigate the mechanisms by which TRIM46 induced cell proliferation, ECAR and OCR, GSEA was performed and results showed that AKT signaling pathway is correlated to TRIM46 expression (Fig. S4G). Overexpression of WT-TRIM46 significantly upregulated the expression of p-AKT (Ser 473), HK2, and GLUT1, which was not seen in the group of RING-mutant TRIM46 overexpression (Fig. 4D, E). While TRIM46 knockdown downregulated the expression of p-AKT (Ser 473), HK2, and GLUT1 in H358 and H1299 cells (Fig. S4H). Glycolysis inhibitor 2-Deoxy-D-glucose (2-DG) significantly ameliorated overexpression of WT-TRIM46 caused an increase of cell proliferation of A549 in the absence or presence of DDP (Fig. S5A, B). Collectively, these results indicated that TRIM46 especially TRIM46’s E3 ligase function promoted LUAD cell proliferation and DDP resistance by enhancing glycolysis.

### TRIM46 promoted PHLPP2 ubiquitination

**Fig. 5:**
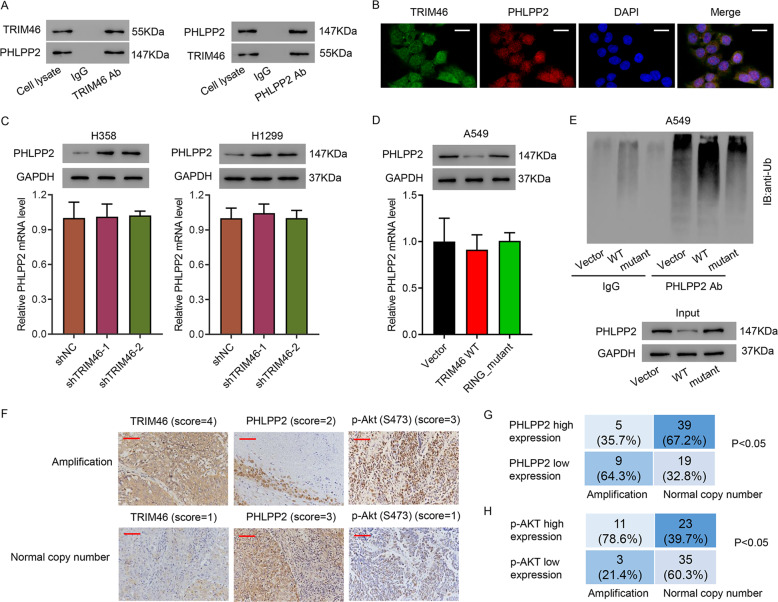
TRIM46 interacted with PHLPP2 and promoted PHLPP2 ubiquitination in LUAD cells. **A** Co-IP assay of TRIM46 and PHLPP2 in H358 cells. **B** Immunofluorescence assay of TRIM46 and PHLPP2 in H358 cells (scale bar: 20 μm). **C** qPCR and immunoblot analysis of PHLPP2 expression in both H358 cells and H1299 cells with TRIM46 silence. **D** Analysis of PHLPP2 expression in A549 cells transfected with WT-TRIM46, RING-mutant TRIM46, or vector. **E** IP analysis of the ubiquitination of PHLPP2 in A549 cells transfected with WT-TRIM46, RING-mutant TRIM46, or vector. **F** Immunohistochemical staining of TRIM46, PHLPP2, and p-AKT in cohort 1 (scale bar: 100 μm). **G**, **H** Statistical analysis of LUAD tissues under different staining conditions in cohort 1. Results were presented as the mean ± standard error (*n* = 3). Independent experiments were repeated three times.

To further study how TRIM46 was involved in LUAD, IP and Mass spectrometry were used to analyze TRIM46 binding proteins (Fig. S6A, B). Results suggested that PHLPP2 was one of the potential TRIM46 binding proteins. Co-IP assay of TRIM46 and PHLPP2 was performed using H358 cell lysates. Co-IP results showed that TRIM46 interacted with PHLPP2 (Fig. 5A). Immunofluorescence staining of TRIM46 and PHLPP2 also confirmed that TRIM46 co-localized with PHLPP2 in H358 cells (Fig. 5B), which was not found in PHLPP2-knockout H358 cells (Fig. S7). Silencing TRIM46 did not affect the mRNA levels of PHLPP2, but significantly increased PHLPP2 at protein level (Fig. 5C). In contrast, overexpressing WT-TRIM46 significantly decreased PHLPP2 at protein level (Fig. 5D). IP analysis of the ubiquitination of PHLPP2 indicated that overexpressing WT-TRIM46 significantly increased the ubiquitination of PHLPP2, but RING-mutant TRIM46 overexpression showed no effect on the ubiquitination of PHLPP2 (Fig. 5E). Then, levels of TRIM46, PHLPP2, and p-AKT in LUAD tissue were measured by immunohistochemical staining. Results suggested that LUAD tissue with TRIM46 amplification increased the levels of TRIM46 and p-AKT, but decreased the levels of PHLPP2 (Fig. 5F). Statistical analysis of LUAD tissues under different staining conditions in cohort 1 indicated that TRIM46 amplification negatively correlated to PHLPP2 level, but positively correlated to p-AKT level (Fig. 5G, H). Together, these data suggest that E3 ligase played a very important role in the interaction between TRIM46 and PHLPP2, and the TRIM46-promoted PHLPP2 ubiquitination in LUAD cells.

### Overexpressing PHLPP2 blocked the effects of TRIM46 overexpression

**Fig. 6:**
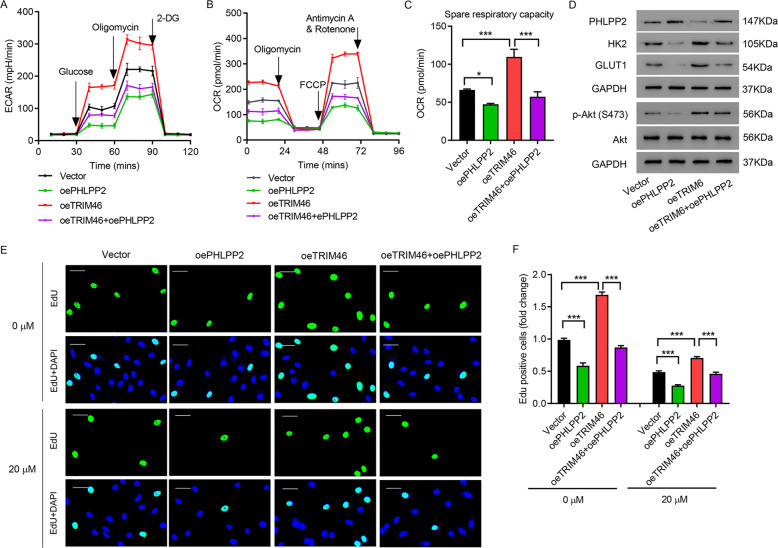
Overexpressing PHLPP2 overexpression abolished the effects of TRIM46 overexpression. **A** ECAR, **B** OCR, **C** spare respiratory capacity and **D** expression of PHLPP2, AKT, p-AKT, HK2, and GLUT1 of A549 cells transfected with TRIM46, PHLPP2, or TRIM46 plus PHLPP2. A549 cells were transfected with PHLPP2/TRIM46 and treated by DDP (20 μM). **E** A549 cell proliferation measured by EdU staining (scale bar: 50 μm). **F** Statistical analysis of EdU positive A549 cells. Results were presented as the mean ± standard error (*n* = 3). Independent experiments were repeated three times. *P < 0.05, ***P < 0.001.

Next, PHLPP2 was overexpressed to further study the role of PHLPP2 in LUAD. Results suggested that overexpression of PHLPP2 significantly ameliorated oeTRIM46-increased ECAR and OCR (Fig. 6A–C). Immunoblotting assay showed that overexpression of PHLPP2 also abolished oeTRIM46-caused decrease of PHLPP2 and increase of p-AKT (Ser 473), HK2, and GLUT1 (Fig. 6D). oeTRIM46-caused DDP resistance in A549 cells was also abolished by overexpression of PHLPP2 (Fig. 6E, F). These findings indicate that the effect of TRIM46 overexpression can be reversed by PHLPP2 overexpression.

### Amplification of TRIM46 promoted PDX growth and enhanced DDP resistance

**Fig. 7:**
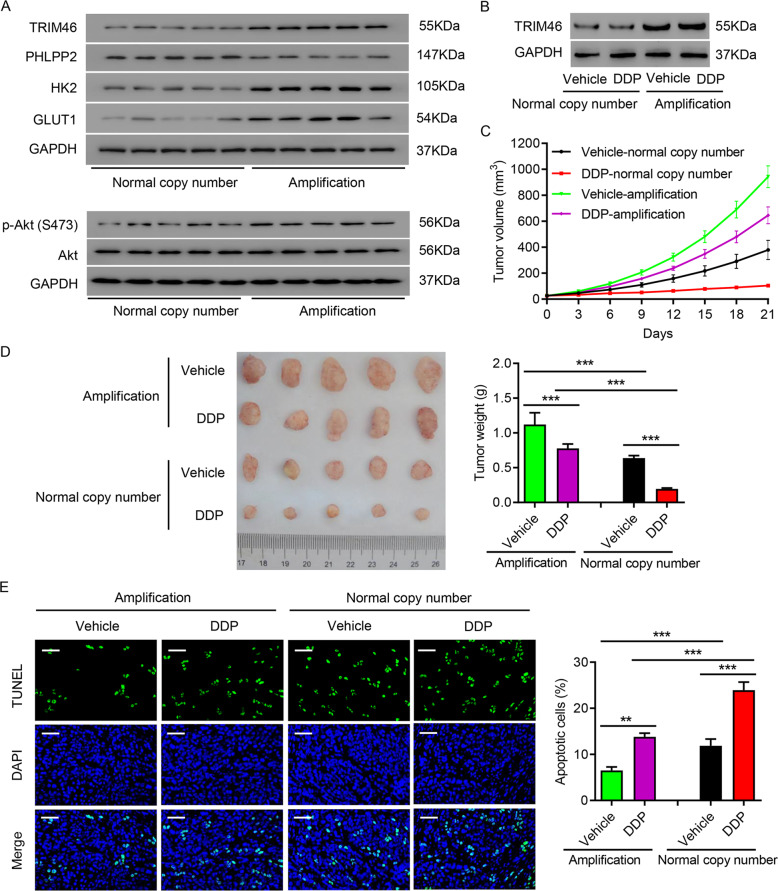
Amplification of TRIM46 promoted PDX growth and enhanced DDP resistance. **A** Expression of PHLPP2, TRIM46, p-AKT (Ser473), HK2, and GLUT1 in LUAD tissues with/without TRIM46 amplification. **B**–**E** LUAD tissues with/without TRIM46 amplification were used to create a PDX model and at seven days after inoculation, DDP (5 mg/kg/d) was administered to tumor-bearing mice by intraperitoneal injection for 3 weeks. **B** Expression of TRIM46 in PDX model with/without TRIM46 amplification. **C** Tumor volume of PDX model. **D** Tumor weight. **E** TUNEL staining results (Scale bar: 50 μm). Results were presented as the mean ± standard error (*n* = 5). Independent experiments were repeated three times. **P < 0.01, ***P < 0.001.

To further explore the relevance of TRIM46 amplification and LUAD, we first measured the expression levels of TRIM46, PHLPP2, AKT, p-AKT (Ser473), HK2, and GLUT1 in lung tumor tissues with and without TRIM46 amplification. Western blots indicated that LUAD tissues with TRIM46 amplification had higher levels of TRIM46, p-AKT, HK2, and GLUT1, but lower levels of PHLPP2 (Fig. 7A). Next, tumor tissues with or without TRIM46 amplification were used to generate a PDX model, and DDP was administered as mentioned above. As shown in Fig. 7B, TRIM46 expression was also increased in the PDXs with TRIM46 amplification. TRIM46 amplification not only increased tumor growth and tumor sizes, but also ameliorated DDP-caused decrease of tumor growth and tumor sizes (Fig. 7C, D). TUNEL staining of tumor tissues revealed that TRIM46 amplification significantly suppressed apoptosis and DDP-induced apoptosis of tumor cells (Fig. 7E). The findings indicated that TRIM46 amplification promoted tumor growth and enhanced its DDP resistance.

## Discussion

In this study, we first analyzed the copy number of 6 TRIM members in both TCGA data and LUAD specimen from our hospital and found that TRIM46 was the one with the highest amplification, and amplification of TRIM46 was significantly associated with a poor survival rate. Therefore, TRIM46 was chosen for further study. TRIM46 expression was shown to be positively correlated with tumor size, TMN stage, and lymphnode metastasis. Our results also support that TRIM46 overexpression increased the proliferation of LUAD cells, promoted xenografts growth, and enhanced DDP resistance of LUAD cells by enhancing glycolysis. Mechanism study indicated that the effects of TRIM46 overexpression in LUAD cells were mediated by TRIM46 ubiquitination of PHLPP2 in LUAD cells because PHLPP2 overexpression reversed the effects of TRIM46 overexpression. Using a PDX model, we further demonstrated that amplification of TRIM46 promoted LUAD growth and enhanced its DDP resistance.

In contrast to normal cells, tumor cells consume a large amount of glucose, and convert most glucose to lactate even in the presence of oxygen [26]. It has been showed that glucose uptake and glycolytic activity were upregulated in lung cancer cells [27]. AKT activation has been shown to be sufficient to promote aerobic glycolysis [28]. Garrido et al. has indicated that activating Akt induced GLUT1 to upregulate glucose uptake [29]. The PI3K/Akt pathway can also contribute to glycolysis by regulating the activity or the expression of some glycolytic effector, such as HK2, one of the five known hexokinase isoforms catalyzes the first committed step in glucose metabolism by phosphorylating glucose [30]. It has been shown that HK2 expression is upregulated by AKT-activation and inhibition of HK2 suppresses lung tumor growth [31]. In this study, we found that TRIM46 overexpression significantly upregulated the expression of p-AKT (Ser 473), which resulted in elevated expression of glycolytic effectors, HK2 and GLUT1, leading to the promotion of glycolysis. These results indicated a new role of TRIM46 amplification in lung cancer. These findings not only increase our knowledge of TRIM46 amplification in glycolysis, but also broaden our understanding of the progression of lung cancer.

Pleckstrin homology domain leucine-rich repeat protein phosphatase 2 (PHLPP2), has been identified as a phosphatase with PH domains [32]. PHLPP proteins have been recognized as tumor suppressors in several types of cancer. For example, PHLPP2 has been shown to inhibit lung carcinogenesis following B[a]P/B[a]PDE exposure [33]. A study by Peng et al. has reported that PHLPP2 stabilization inhibits bladder cancer invasion by promoting autophagic degradation of MMP2 protein [34]. PHLPP2 has also been shown to play a central role in AKT signaling modulation [35]. PHLPP removes the phosphor-group form serine residues to inhibit AKT [35, 36]. Hribal et al. has shown that increased PHLPP2 and PHLPP1 were paralleled by decreased phosphorylation of AKT. Strotbek et al. has indicated that suppressing the expression of PHLPP2 results in elevated growth factor-induced AKT phosphorylation [37]. In the current study, we proved that TRIM46 interacted with PHLPP2 and promoted PHLPP2 ubiquitination in LUAD cells. These findings revealed a new role of PHLPP2 in lung cancer, showing that LUAD tissue with TRIM46 amplification had decreased levels of PHLPP2, leading to increased levels of p-AKT which promotes glycolysis. In addition to PHLPP2, one of the protein-protein interactions identified in the mass spectrometry experiment was EIF3A (one of the RNA-binding components) that is required for the initiation of protein synthesis, and its expression was associated with the response of lung cancer patients to platinum-based chemotherapy through the regulation of DNA repair pathways [38]. Therefore, more studies of other TRIM46 targets that promote cancer growth are needed in this area.

Chemo-resistance is one of the major concerns in cancer treatment [39]. According to drug responsiveness, drug resistance can be classified into intrinsic resistance and acquired resistance [40]. The mechanisms of chemoresistance are complicated [41]. Higher level of p-AKT has been shown in paclitaxel-resistant cancer cells [42]. Another study indicated that inhibiting AKT alleviates the chemoresistance of cancer cells via decreasing cancer stem cell marker [43]. It also has been reported that a scaffolding protein of PHLPP, FKBP51, affects the chemosensitivity of cancer cells by enhancing PHLPP-mediated dephosphorylation of AKT [44]. Our results demonstrated that overexpression of PHLPP2 abolished oeTRIM46-caused downregulation of PHLPP2 and upregulation of p-AKT (Ser 473), and diminished oeTRIM46-caused inhibition of apoptosis in LUAD cells. These findings indicate a very important role of TRIM46/PHLPP2/AKT in regulating chemosensitivity and improve our understanding of the chemoresistance in cancer treatment. There are certainly some limitations. To further elucidate the function of TRIM46/ PHLPP2/AKT in lung cancer, an orthotopic mouse model needs to be employed in later research. Further studies using PHLPP2 activators in PDX animal models will provide more relevant data. Although further studies are needed, this study identifies a new molecular mechanism underlying chemoresistance of lung cancer.

**Fig. 8:**
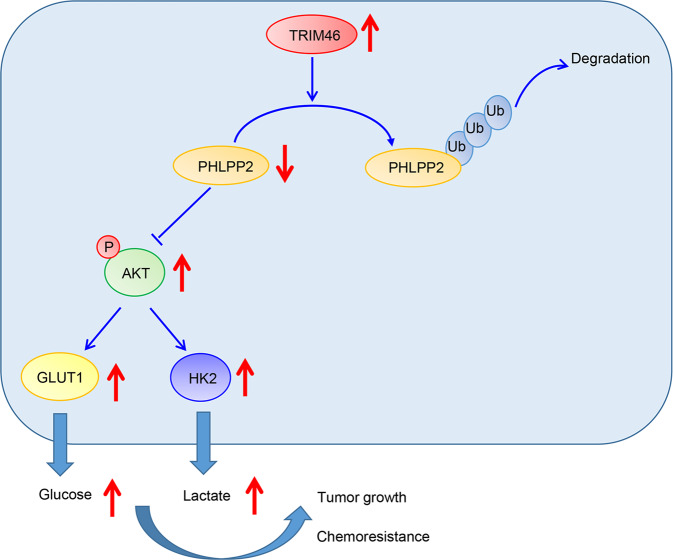
Depicting the possible molecular mechanisms by which TRIM46 regulates LUAD progression. TRIM46 activates AKT/HK2 signaling by modifying PHLPP2 ubiquitylation to promote glycolysis and chemoresistance of lung cancer cells.

In conclusion, our study revealed a new role of TRIM46/PHLPP2/AKT signaling, showing that TRIM46 overexpression increased the proliferation of LUAD cells, promoted xenografts growth, and enhanced DDP resistance of LUAD cells by enhancing glycolysis through activation of AKT/HK2 signaling (Fig. 8). The findings highlighted the importance of TRIM46/PHLPP2/AKT signaling which might be helpful in developing new drugs for lung cancer treatments.

## Supplementary information

Blots

Supplemental data

aj-checklist

## Data Availability

All data generated or analyzed during this study are included in this published article (and its Supplementary Information files).
