# Supplementary material for: TRIM46 activates AKT/HK2 signaling by modifying PHLPP2 ubiquitylation to promote glycolysis and chemoresistance of lung cancer cells
Source: Cell Death Dis. 2022 Mar 30;13(3):285. doi: 10.1038/s41419-022-04727-7 (PMC8967906; doi:10.1038/s41419-022-04727-7)

Figure 4D

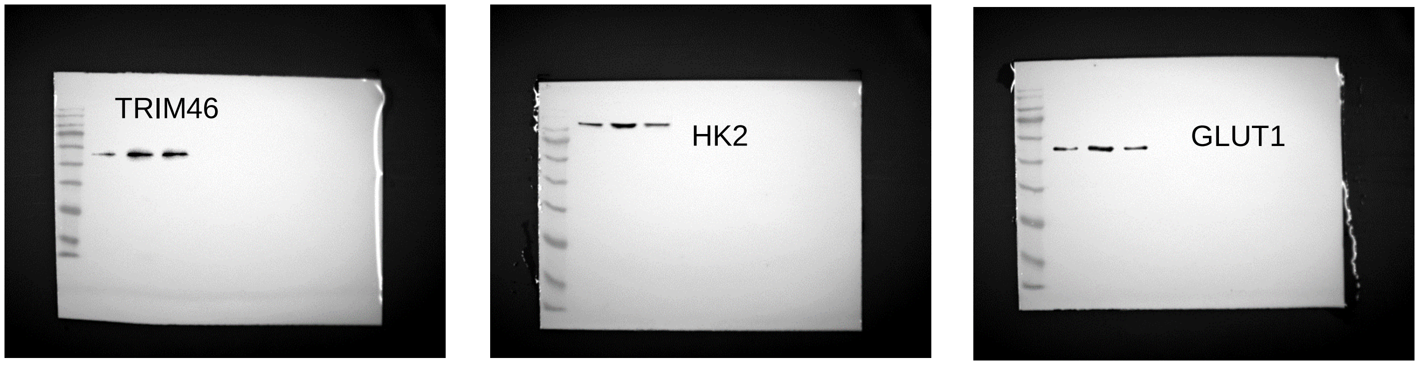

Figure 4D

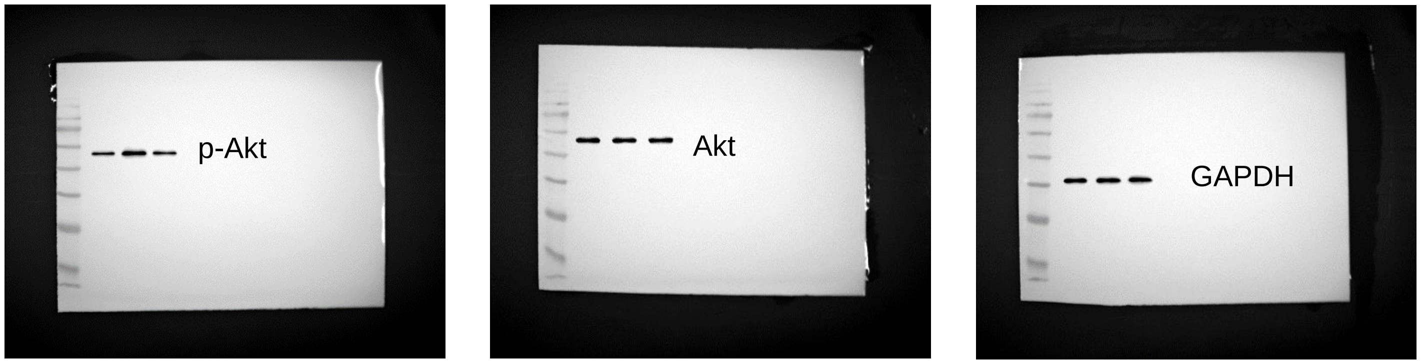

Figure 5A left

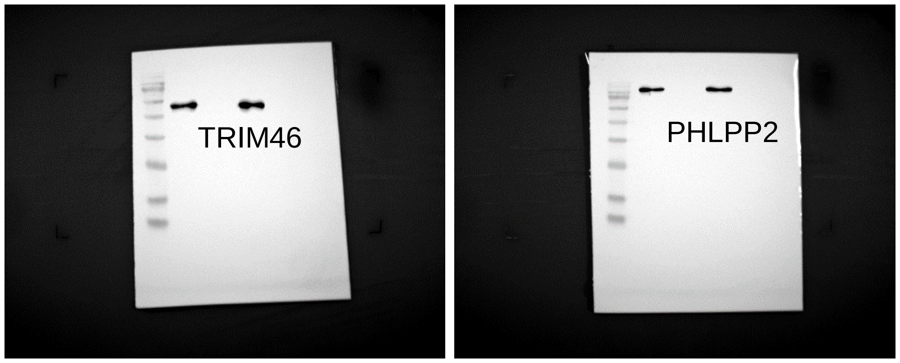

Figure 5A right

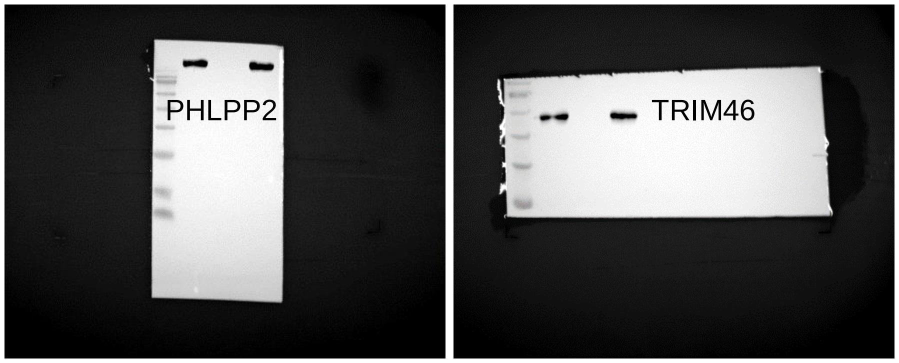

Figure 5C H358

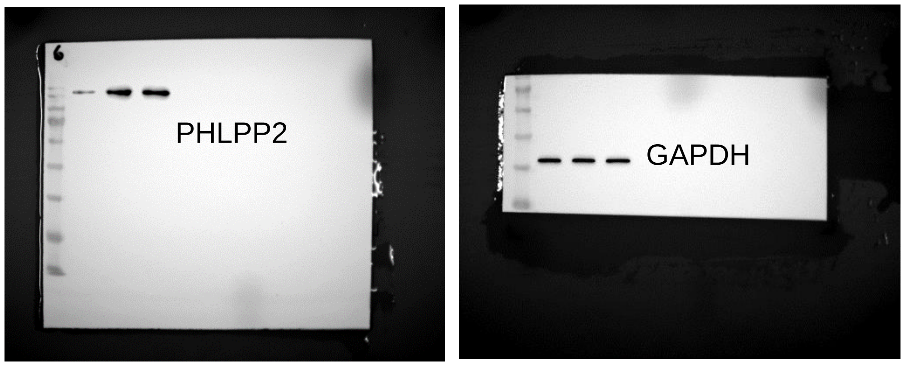

Figure 5C H1299

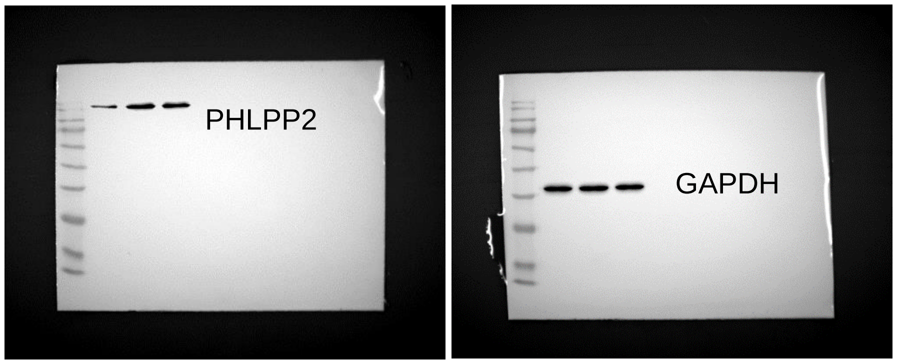

Figure 5D

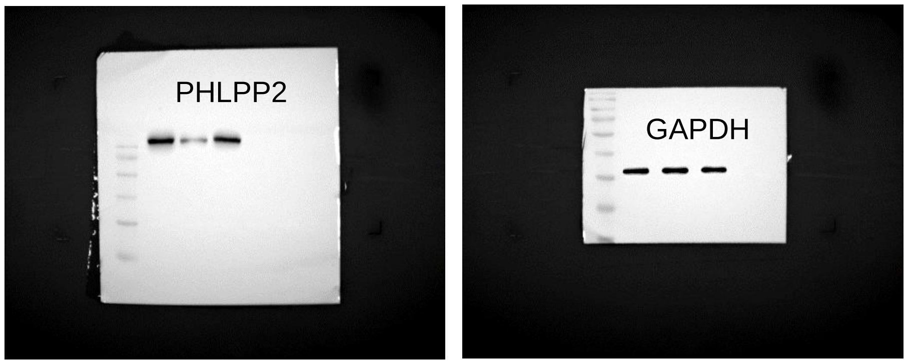

Figure 5E

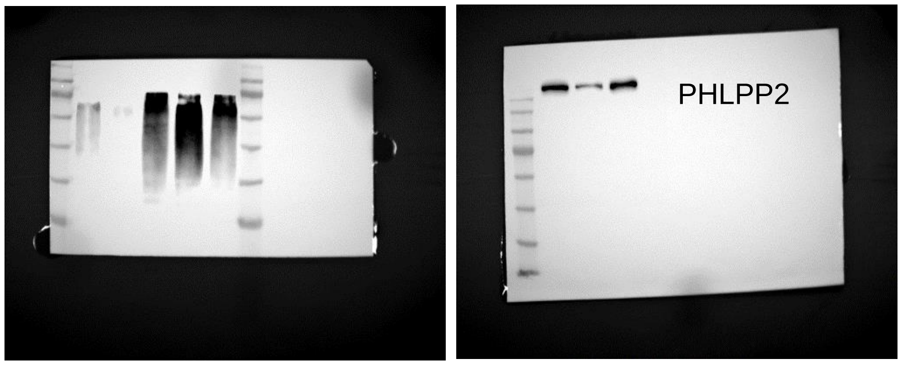

Figure 5E

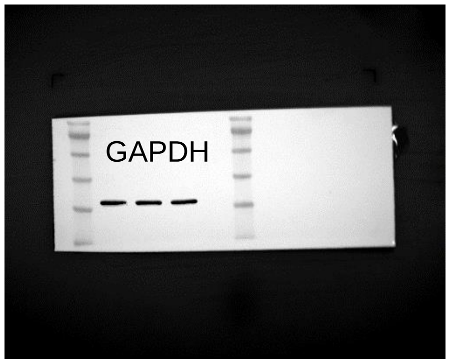

Figure 6D

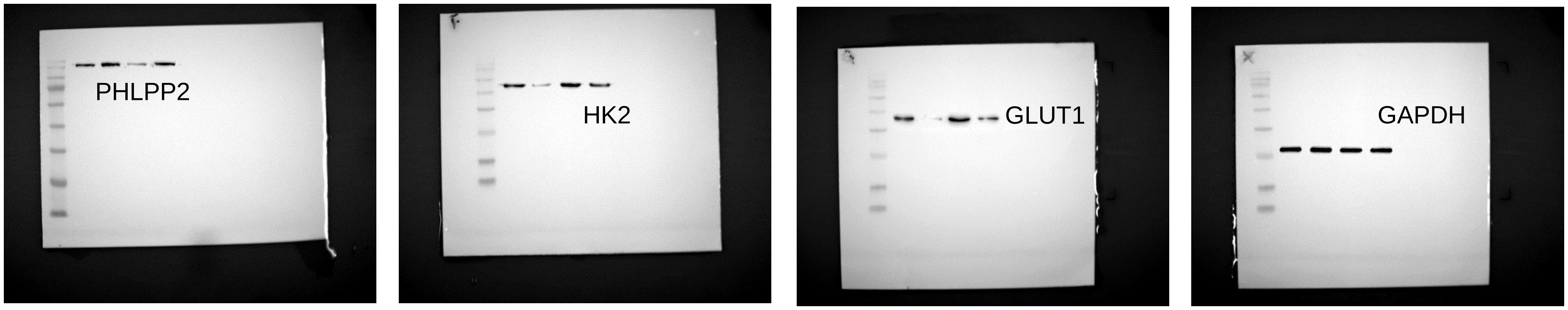

Figure 6D

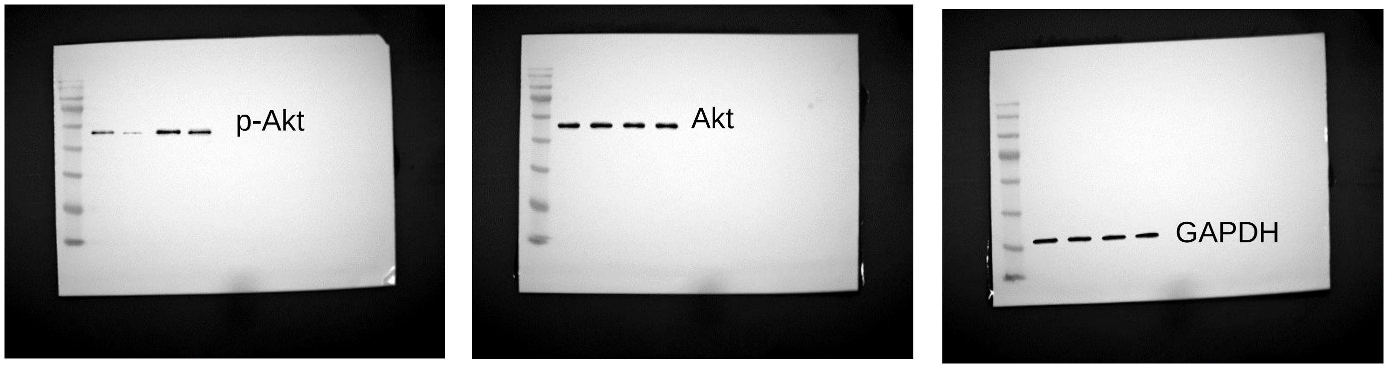

Figure 7A

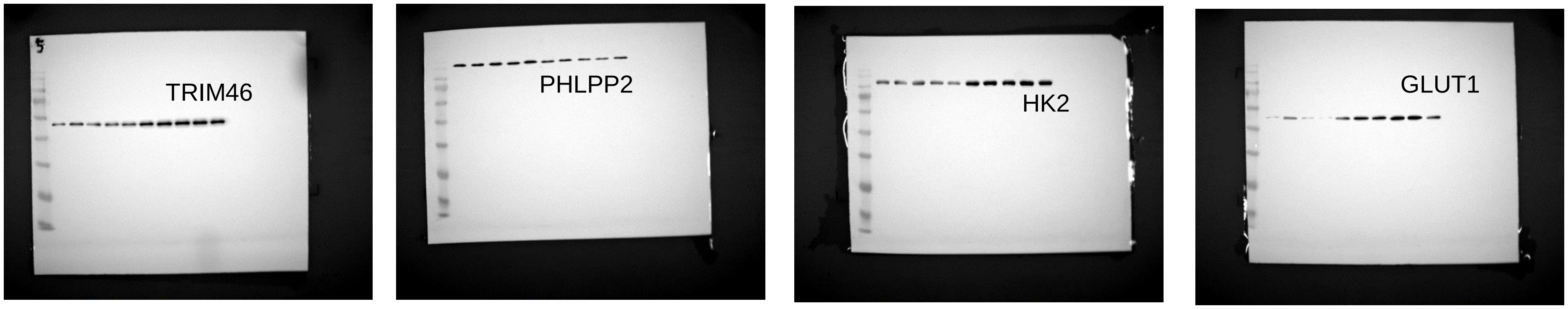

Figure 7A

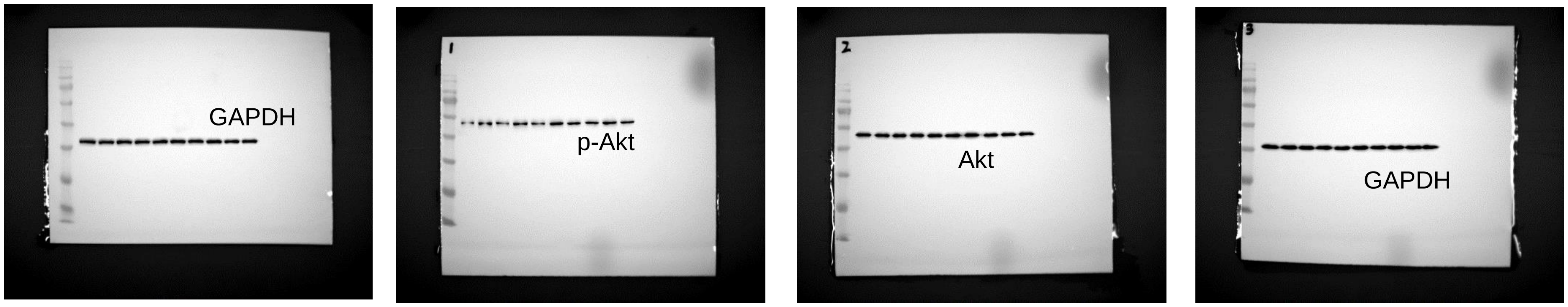

Figure 7B

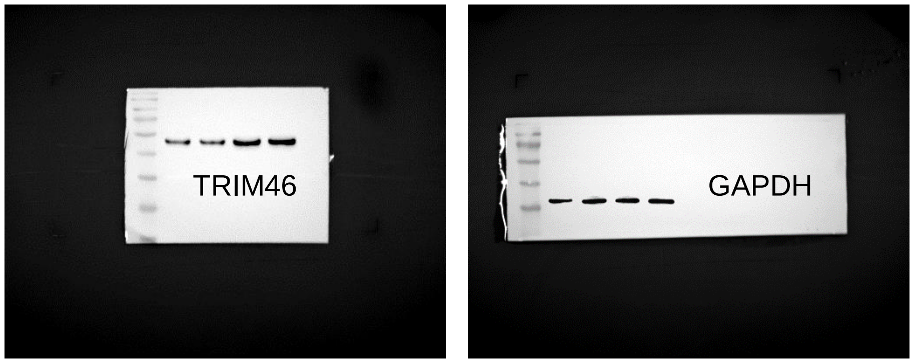

Figure S1A

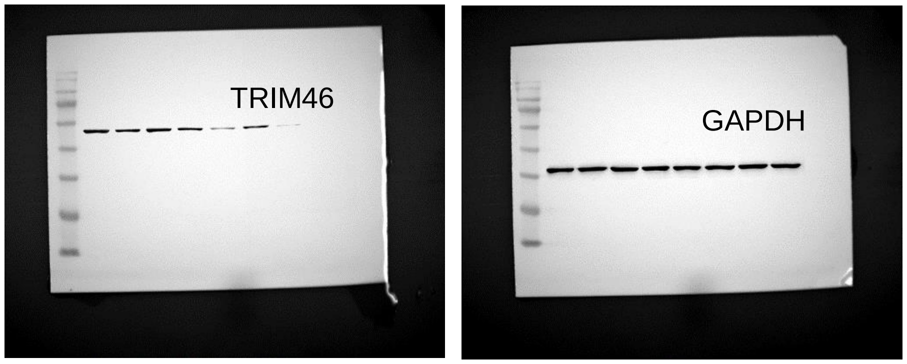

Figure S1B

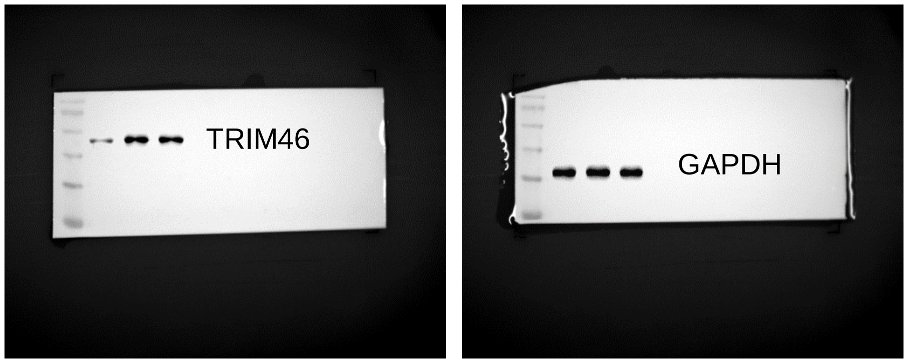

Figure S3A
H358

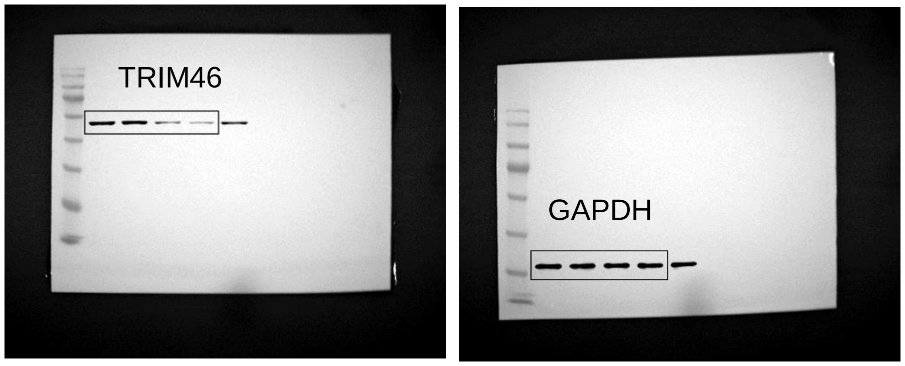

Figure S3A
H1299

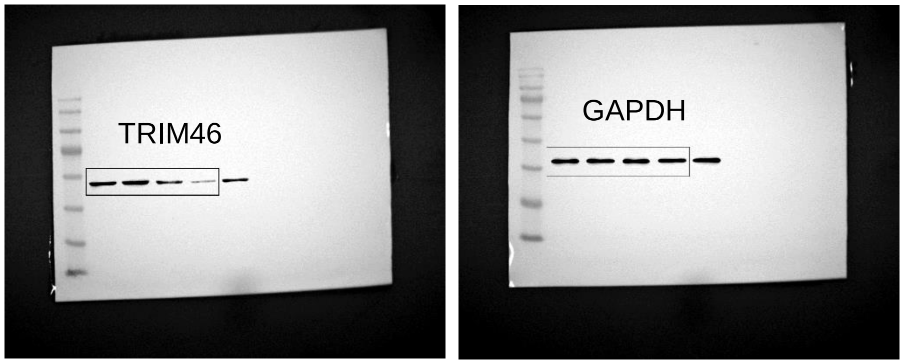

Figure S4H
H358

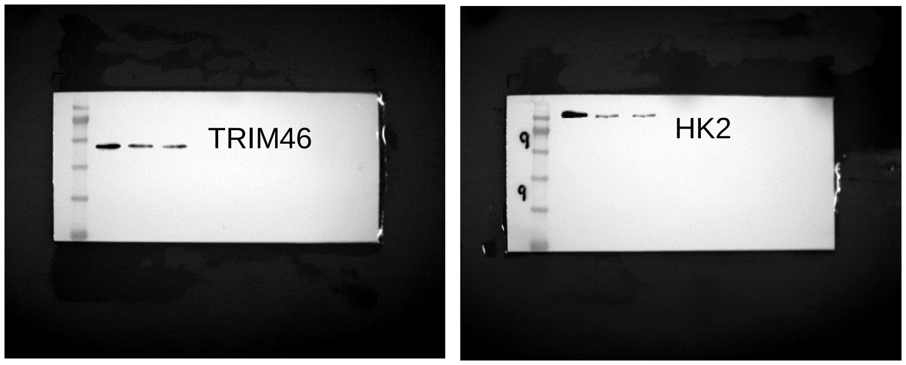

Figure S4H
H1299

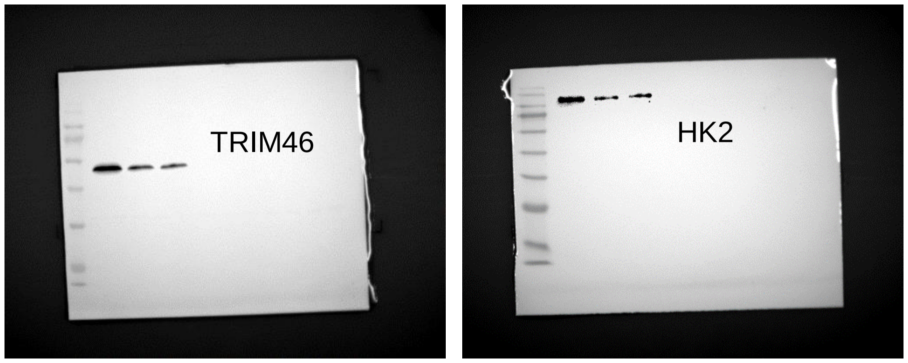

Figure S4H
H358

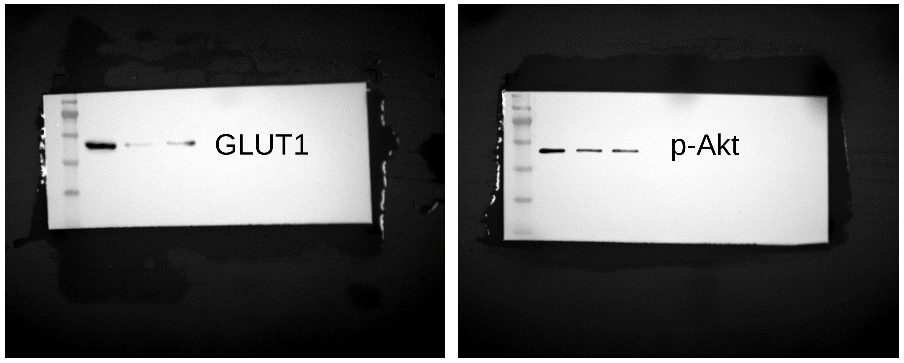

Figure S4H
H1299

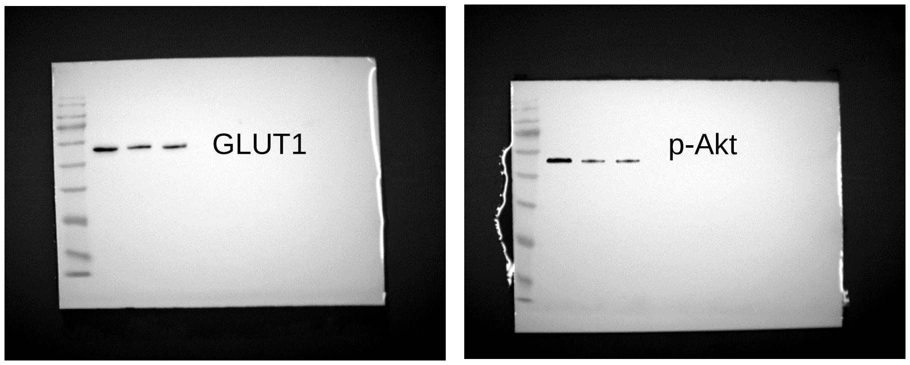

Figure S4H
H358

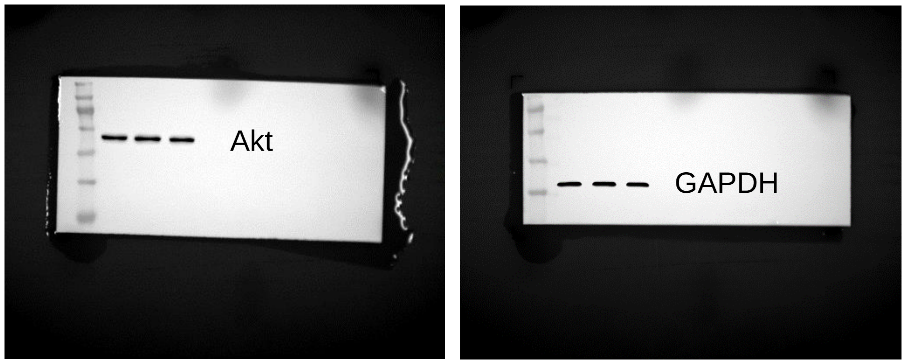

Figure S4H
H1299

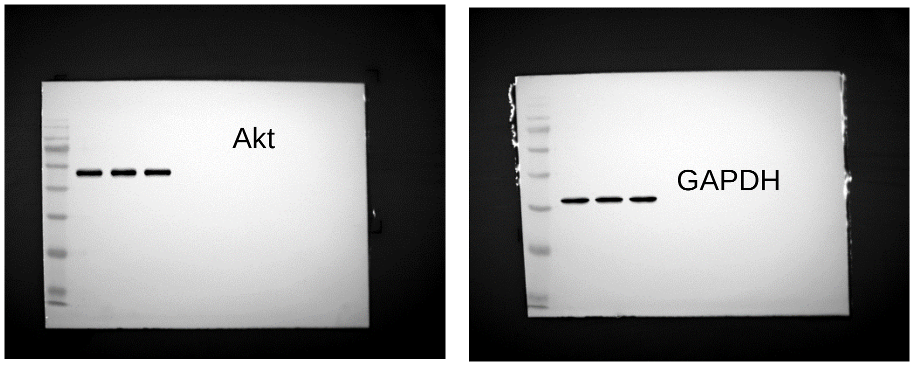

Supplement: Supplementary file 1 — Blots [file 41419_2022_4727_MOESM1_ESM.pdf]
