# Supplementary material for: TRIM46 activates AKT/HK2 signaling by modifying PHLPP2 ubiquitylation to promote glycolysis and chemoresistance of lung cancer cells
Source: Cell Death Dis. 2022 Mar 30;13(3):285. doi: 10.1038/s41419-022-04727-7 (PMC8967906; doi:10.1038/s41419-022-04727-7)
Supplement: Supplementary file 2 — Supplemental data [file 41419_2022_4727_MOESM2_ESM.docx]

**TRIM46 activates AKT/HK2 signaling by modifying PHLPP2 ubiquitylation to promote glycolysis and chemoresistance of lung cancer cells**

**Running title:** TRIM46 and lung cancer

Jicheng Tantai^*^, Xufeng Pan^*^, Yong Chen, Yuzhou Shen, Chunyu Ji^#^

Department of Thoracic Surgery, Shanghai Chest Hospital, Shanghai Jiao Tong University, Shanghai, China

^*^Contributed equally

^#^Corresponding to: Dr. Chunyu Ji, Address: No.241 West Huaihai Road, Shanghai 200030, China; Tel: 86-21-62821990; Email: 13601870843@163.com

**Supplemental material**

**Immunoprecipitation (IP) and LC/MS analysis**

Proteins of H358 cells were extracted, pre-cleared with IgG and protein A/G beads, and incubated with anti-TRIM46 or control IgG overnight. The immunoprecipitated proteins were separated, and stained with Coomassie Brilliant Blue. Several bands were excised, digested, and analyzed.

**Table S1.** Antibody list.

| **Primary antibody** | **Company** | **Catalog No.** |
| --- | --- | --- |
| TRIM46 | Abcam | Ab169044 |
| PHLPP2 | Abcam | Ab153918 |
| HK2 | Abcam | Ab209847 |
| GLUT1 | Cell Signaling Technology | #12939 |
| p-AKT(S473) | Cell Signaling Technology | #9271 |
| AKT | Cell Signaling Technology | #9272 |
| GAPDH | Cell Signaling Technology | #5174 |
| Ubiquitin | Abcam | Ab7780 |

**Table S2.** List of proteins identified by mass spectrometry.

| Accession | Description | Score | Coverage | # Proteins | # Unique Peptides | # Peptides | # PSMs | # AAs | MW [kDa] | calc. pI |
| --- | --- | --- | --- | --- | --- | --- | --- | --- | --- | --- |
| Q7Z4K8 | Tripartite Motif Containing 46 TRIM46 OS=Homo sapiens GN=TRIM46 PE=1 SV=2 - [TRIM46_HUMAN] | 88.50 | 5.65 | 1 | 8 | 9 | 9 | 5890 | 83.4 | 7.75 |
| P20930 | Filaggrin OS=Homo sapiens GN=FLG PE=1 SV=3 - [FILA_HUMAN] | 55.95 | 1.77 | 1 | 1 | 1 | 1 | 4061 | 434.9 | 9.25 |
| Q6ZVD8 | PH Domain And Leucine Rich Repeat Protein Phosphatase 2 OS=Homo sapiens GN=PHLPP2 PE=1 SV=2 - [PHLPP2_HUMAN] | 0.00 | 0.42 | 1 | 1 | 1 | 1 | 2364 | 146.8 | 5.48 |
| Q8IWZ3 | Ankyrin repeat and KH domain-containing protein 1 OS=Homo sapiens GN=ANKHD1 PE=1 SV=1 - [ANKH1_HUMAN] | 30.08 | 0.28 | 1 | 1 | 1 | 1 | 2542 | 269.3 | 5.73 |
| Q9P219 | Protein Daple OS=Homo sapiens GN=CCDC88C PE=1 SV=3 - [DAPLE_HUMAN] | 18.58 | 0.49 | 1 | 1 | 1 | 1 | 2028 | 228.1 | 6.23 |
| P35579 | Myosin-9 OS=Homo sapiens GN=MYH9 PE=1 SV=4 - [MYH9_HUMAN] | 77.27 | 3.27 | 1 | 5 | 5 | 5 | 1960 | 226.4 | 5.60 |
| O75051 | Plexin-A2 OS=Homo sapiens GN=PLXNA2 PE=1 SV=4 - [PLXA2_HUMAN] | 26.87 | 0.32 | 1 | 1 | 1 | 1 | 1894 | 211.0 | 6.48 |
| Q9BVV6 | Protein TALPID3 OS=Homo sapiens GN=KIAA0586 PE=1 SV=4 - [TALD3_HUMAN] | 22.18 | 0.65 | 1 | 1 | 1 | 1 | 1533 | 169.2 | 5.54 |
| Q14152 | Eukaryotic translation initiation factor 3 subunit A OS=Homo sapiens GN=EIF3A PE=1 SV=1 - [EIF3A_HUMAN] | 28.31 | 1.81 | 1 | 2 | 2 | 2 | 1382 | 166.5 | 6.79 |
| O75064 | DENN domain-containing protein 4B OS=Homo sapiens GN=DENND4B PE=1 SV=4 - [DEN4B_HUMAN] | 22.14 | 0.40 | 3 | 1 | 1 | 1 | 1496 | 163.7 | 7.21 |
| O75533 | Splicing factor 3B subunit 1 OS=Homo sapiens GN=SF3B1 PE=1 SV=3 - [SF3B1_HUMAN] | 30.40 | 1.46 | 1 | 1 | 1 | 1 | 1304 | 145.7 | 7.09 |
| Q9NQC3 | Reticulon-4 OS=Homo sapiens GN=RTN4 PE=1 SV=2 - [RTN4_HUMAN] | 348.37 | 9.48 | 1 | 7 | 7 | 16 | 1192 | 129.9 | 4.50 |
| P27816 | Microtubule-associated protein 4 OS=Homo sapiens GN=MAP4 PE=1 SV=3 - [MAP4_HUMAN] | 106.04 | 7.03 | 1 | 6 | 6 | 6 | 1152 | 120.9 | 5.43 |
| Q02413 | Desmoglein-1 OS=Homo sapiens GN=DSG1 PE=1 SV=2 - [DSG1_HUMAN] | 88.32 | 3.34 | 1 | 2 | 2 | 2 | 1049 | 113.7 | 5.03 |
| Q9Y5I4 | Protocadherin alpha-C2 OS=Homo sapiens GN=PCDHAC2 PE=2 SV=1 - [PCDC2_HUMAN] | 42.77 | 0.70 | 1 | 1 | 1 | 1 | 1007 | 109.4 | 5.41 |
| Q9Y2W1 | Thyroid hormone receptor-associated protein 3 OS=Homo sapiens GN=THRAP3 PE=1 SV=2 - [TR150_HUMAN] | 33.96 | 5.45 | 1 | 3 | 3 | 3 | 955 | 108.6 | 10.15 |
| P22102 | Trifunctional purine biosynthetic protein adenosine-3 OS=Homo sapiens GN=GART PE=1 SV=1 - [PUR2_HUMAN] | 31.66 | 2.97 | 1 | 3 | 3 | 3 | 1010 | 107.7 | 6.70 |
| Q9NYF8 | Bcl-2-associated transcription factor 1 OS=Homo sapiens GN=BCLAF1 PE=1 SV=2 - [BCLF1_HUMAN] | 24.92 | 1.30 | 1 | 1 | 1 | 1 | 920 | 106.1 | 9.98 |
| P12814 | Alpha-actinin-1 OS=Homo sapiens GN=ACTN1 PE=1 SV=2 - [ACTN1_HUMAN] | 50.21 | 1.35 | 2 | 1 | 1 | 1 | 892 | 103.0 | 5.41 |
| Q13435 | Splicing factor 3B subunit 2 OS=Homo sapiens GN=SF3B2 PE=1 SV=2 - [SF3B2_HUMAN] | 25.18 | 2.35 | 1 | 2 | 2 | 2 | 895 | 100.2 | 5.67 |
| Q08554 | Desmocollin-1 OS=Homo sapiens GN=DSC1 PE=1 SV=2 - [DSC1_HUMAN] | 27.22 | 3.36 | 1 | 2 | 2 | 2 | 894 | 99.9 | 5.43 |
| P14625 | Endoplasmin OS=Homo sapiens GN=HSP90B1 PE=1 SV=1 - [ENPL_HUMAN] | 38.75 | 3.49 | 1 | 2 | 2 | 2 | 803 | 92.4 | 4.84 |
| Q00839 | Heterogeneous nuclear ribonucleoprotein U OS=Homo sapiens GN=HNRNPU PE=1 SV=6 - [HNRPU_HUMAN] | 62.91 | 7.27 | 1 | 3 | 3 | 3 | 825 | 90.5 | 6.00 |
| Q674X7 | Kazrin OS=Homo sapiens GN=KAZN PE=1 SV=2 - [KAZRN_HUMAN] | 34.79 | 1.55 | 1 | 1 | 1 | 1 | 775 | 86.3 | 7.03 |
| Q12797 | Aspartyl/asparaginyl beta-hydroxylase OS=Homo sapiens GN=ASPH PE=1 SV=3 - [ASPH_HUMAN] | 183.07 | 6.86 | 1 | 5 | 5 | 6 | 758 | 85.8 | 5.01 |
| P49321 | Nuclear autoantigenic sperm protein OS=Homo sapiens GN=NASP PE=1 SV=2 - [NASP_HUMAN] | 0.00 | 1.02 | 1 | 1 | 1 | 1 | 788 | 85.2 | 4.30 |
| P07900 | Heat shock protein HSP 90-alpha OS=Homo sapiens GN=HSP90AA1 PE=1 SV=5 - [HS90A_HUMAN] | 110.54 | 6.42 | 4 | 1 | 4 | 5 | 732 | 84.6 | 5.02 |
| P01833 | Polymeric immunoglobulin receptor OS=Homo sapiens GN=PIGR PE=1 SV=4 - [PIGR_HUMAN] | 64.06 | 2.09 | 1 | 1 | 1 | 1 | 764 | 83.2 | 5.74 |
| P08238 | Heat shock protein HSP 90-beta OS=Homo sapiens GN=HSP90AB1 PE=1 SV=4 - [HS90B_HUMAN] | 172.35 | 14.09 | 4 | 5 | 8 | 9 | 724 | 83.2 | 5.03 |
| Q7Z2D5 | Lipid phosphate phosphatase-related protein type 4 OS=Homo sapiens GN=LPPR4 PE=1 SV=1 - [LPPR4_HUMAN] | 40.71 | 1.05 | 1 | 1 | 1 | 1 | 763 | 82.9 | 8.73 |
| Q9Y608 | Leucine-rich repeat flightless-interacting protein 2 OS=Homo sapiens GN=LRRFIP2 PE=1 SV=1 - [LRRF2_HUMAN] | 163.62 | 7.63 | 1 | 4 | 4 | 5 | 721 | 82.1 | 6.95 |
| Q13061 | Triadin OS=Homo sapiens GN=TRDN PE=1 SV=4 - [TRDN_HUMAN] | 47.94 | 1.37 | 1 | 1 | 1 | 1 | 729 | 81.5 | 9.42 |
| Q96NL6 | Sodium channel and clathrin linker 1 OS=Homo sapiens GN=SCLT1 PE=1 SV=2 - [SCLT1_HUMAN] | 28.89 | 1.02 | 1 | 1 | 1 | 1 | 688 | 80.9 | 6.07 |
| Q9H6S3 | Epidermal growth factor receptor kinase substrate 8-like protein 2 OS=Homo sapiens GN=EPS8L2 PE=1 SV=2 - [ES8L2_HUMAN] | 35.06 | 1.54 | 1 | 1 | 1 | 1 | 715 | 80.6 | 6.84 |
| P02788 | Lactotransferrin OS=Homo sapiens GN=LTF PE=1 SV=6 - [TRFL_HUMAN] | 36.31 | 1.27 | 1 | 1 | 1 | 1 | 710 | 78.1 | 8.12 |
| P52272 | Heterogeneous nuclear ribonucleoprotein M OS=Homo sapiens GN=HNRNPM PE=1 SV=3 - [HNRPM_HUMAN] | 33.63 | 2.05 | 1 | 1 | 1 | 1 | 730 | 77.5 | 8.70 |
| P0CG48 | Polyubiquitin-C OS=Homo sapiens GN=UBC PE=1 SV=3 - [UBC_HUMAN] | 156.60 | 72.26 | 4 | 6 | 6 | 8 | 685 | 77.0 | 7.66 |
| Q08188 | Protein-glutamine gamma-glutamyltransferase E OS=Homo sapiens GN=TGM3 PE=1 SV=4 - [TGM3_HUMAN] | 50.63 | 2.89 | 1 | 1 | 1 | 1 | 693 | 76.6 | 5.86 |
| P19338 | Nucleolin OS=Homo sapiens GN=NCL PE=1 SV=3 - [NUCL_HUMAN] | 153.56 | 14.51 | 1 | 8 | 8 | 10 | 710 | 76.6 | 4.70 |
| O15234 | Protein CASC3 OS=Homo sapiens GN=CASC3 PE=1 SV=2 - [CASC3_HUMAN] | 40.56 | 1.56 | 1 | 1 | 1 | 1 | 703 | 76.2 | 6.48 |
| P23246 | Splicing factor, proline- and glutamine-rich OS=Homo sapiens GN=SFPQ PE=1 SV=2 - [SFPQ_HUMAN] | 50.10 | 3.54 | 1 | 1 | 2 | 2 | 707 | 76.1 | 9.44 |
| Q15582 | Transforming growth factor-beta-induced protein ig-h3 OS=Homo sapiens GN=TGFBI PE=1 SV=1 - [BGH3_HUMAN] | 0.00 | 1.90 | 1 | 1 | 1 | 1 | 683 | 74.6 | 7.71 |
| P02545 | Prelamin-A/C OS=Homo sapiens GN=LMNA PE=1 SV=1 - [LMNA_HUMAN] | 193.30 | 12.05 | 1 | 7 | 7 | 8 | 664 | 74.1 | 7.02 |
| O94925 | Glutaminase kidney isoform, mitochondrial OS=Homo sapiens GN=GLS PE=1 SV=1 - [GLSK_HUMAN] | 345.29 | 20.93 | 1 | 13 | 13 | 21 | 669 | 73.4 | 7.77 |
| Q96B97 | SH3 domain-containing kinase-binding protein 1 OS=Homo sapiens GN=SH3KBP1 PE=1 SV=2 - [SH3K1_HUMAN] | 41.89 | 10.98 | 1 | 5 | 5 | 5 | 665 | 73.1 | 6.62 |
| O43390 | Heterogeneous nuclear ribonucleoprotein R OS=Homo sapiens GN=HNRNPR PE=1 SV=1 - [HNRPR_HUMAN] | 36.64 | 3.32 | 1 | 1 | 2 | 2 | 633 | 70.9 | 8.13 |
| Q16881 | Thioredoxin reductase 1, cytoplasmic OS=Homo sapiens GN=TXNRD1 PE=1 SV=3 - [TRXR1_HUMAN] | 221.54 | 14.18 | 1 | 7 | 7 | 10 | 649 | 70.9 | 7.39 |
| P11142 | Heat shock cognate 71 kDa protein OS=Homo sapiens GN=HSPA8 PE=1 SV=1 - [HSP7C_HUMAN] | 107.80 | 10.06 | 2 | 5 | 5 | 7 | 646 | 70.9 | 5.52 |
| O60506 | Heterogeneous nuclear ribonucleoprotein Q OS=Homo sapiens GN=SYNCRIP PE=1 SV=2 - [HNRPQ_HUMAN] | 73.42 | 5.30 | 1 | 2 | 3 | 3 | 623 | 69.6 | 8.59 |
| P02768 | Serum albumin OS=Homo sapiens GN=ALB PE=1 SV=2 - [ALBU_HUMAN] | 176.82 | 7.39 | 1 | 5 | 5 | 8 | 609 | 69.3 | 6.28 |
| O76031 | ATP-dependent Clp protease ATP-binding subunit clpX-like, mitochondrial OS=Homo sapiens GN=CLPX PE=1 SV=2 - [CLPX_HUMAN] | 34.60 | 2.05 | 1 | 1 | 1 | 1 | 633 | 69.2 | 7.58 |
| P23588 | Eukaryotic translation initiation factor 4B OS=Homo sapiens GN=EIF4B PE=1 SV=2 - [IF4B_HUMAN] | 38.64 | 7.04 | 1 | 3 | 3 | 3 | 611 | 69.1 | 5.73 |
| Q01844 | RNA-binding protein EWS OS=Homo sapiens GN=EWSR1 PE=1 SV=1 - [EWS_HUMAN] | 37.44 | 5.95 | 1 | 2 | 2 | 2 | 656 | 68.4 | 9.33 |
| P26038 | Moesin OS=Homo sapiens GN=MSN PE=1 SV=3 - [MOES_HUMAN] | 33.98 | 1.56 | 3 | 1 | 1 | 1 | 577 | 67.8 | 6.40 |
| Q96AE4 | Far upstream element-binding protein 1 OS=Homo sapiens GN=FUBP1 PE=1 SV=3 - [FUBP1_HUMAN] | 27.87 | 1.55 | 1 | 1 | 1 | 1 | 644 | 67.5 | 7.61 |
| Q5JTV8 | Torsin-1A-interacting protein 1 OS=Homo sapiens GN=TOR1AIP1 PE=1 SV=2 - [TOIP1_HUMAN] | 46.21 | 9.95 | 1 | 4 | 4 | 4 | 583 | 66.2 | 8.18 |

**
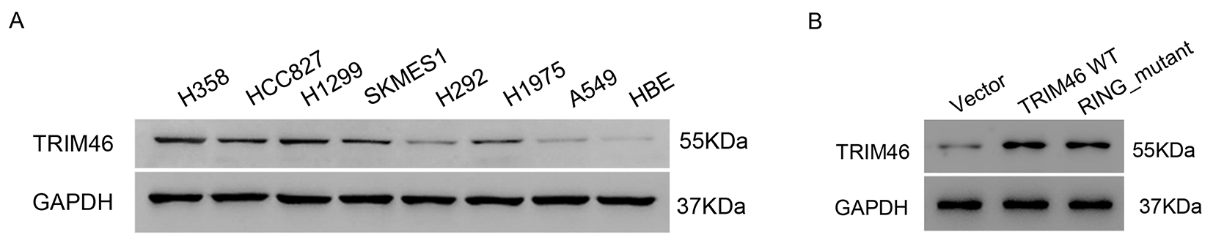
**

**Figure S1.** TRIM46 expression in LUAD cell lines. (A) TRIM46 protein expression in LUAD cell lines. (B) Western blot analysis of TRIM46 expression in A549 cells transfected with WT- or RING-mutant TRIM46 vector.


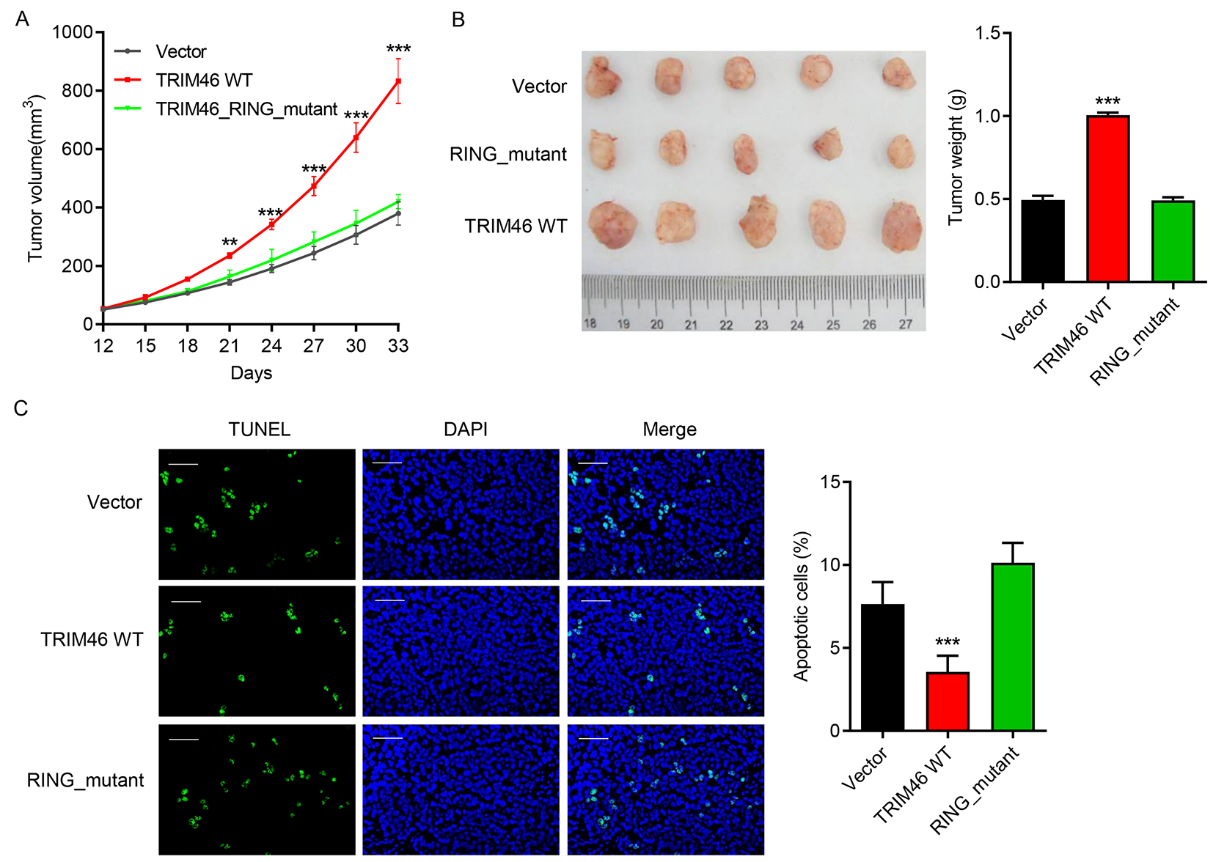


**Figure S2.** TRIM46 overexpression promoted LUAD cell growth in vivo. A549 cells stably expressing TRIM46 (TRIM46 WT), RING-mutant, or Vector control were injected subcutaneously to nude mice. (A) Tumor growth curve. (B) Tumor weight. (C) TUNEL staining results (Scale bar: 50 μm). Results were presented as the mean ± standard error (*n* = 5). Independent experiments were repeated three times. **P<0.01, ***P<0.001 vs. Vector.


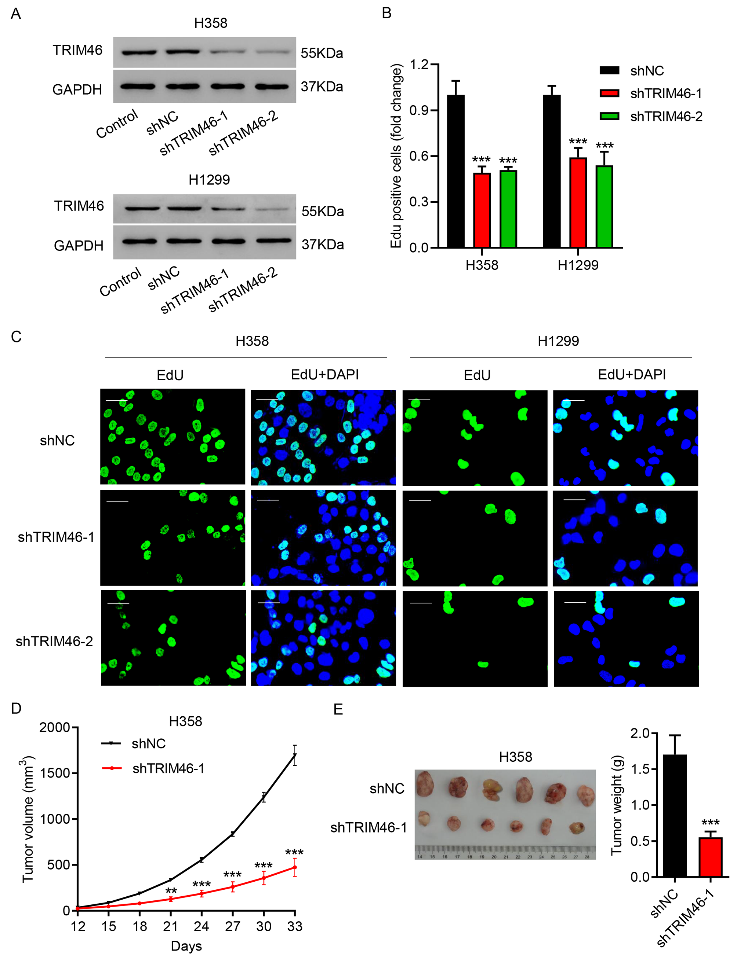


**Figure S3.** TRIM46 silencing inhibited LUAD cell growth in vitro and in vivo. (A) TRIM46 was successfully silenced in H358 and H1299 cells. (B, C) Silencing TRIM46 significantly suppressed the growth of H358 and H1299 cells (Scale bar: 50 μm). Results were presented as the mean ± standard error (*n* = 3). H358 cells stably expressing TRIM46 shRNA (shTRIM46-1) or shNC control were injected subcutaneously to nude mice (n=6). (D) Tumor growth curve. (E) Tumor weight. Results were presented as the mean ± standard error (*n* = 6). Independent experiments were repeated three times. **P<0.01, ***P<0.001vs shNC.


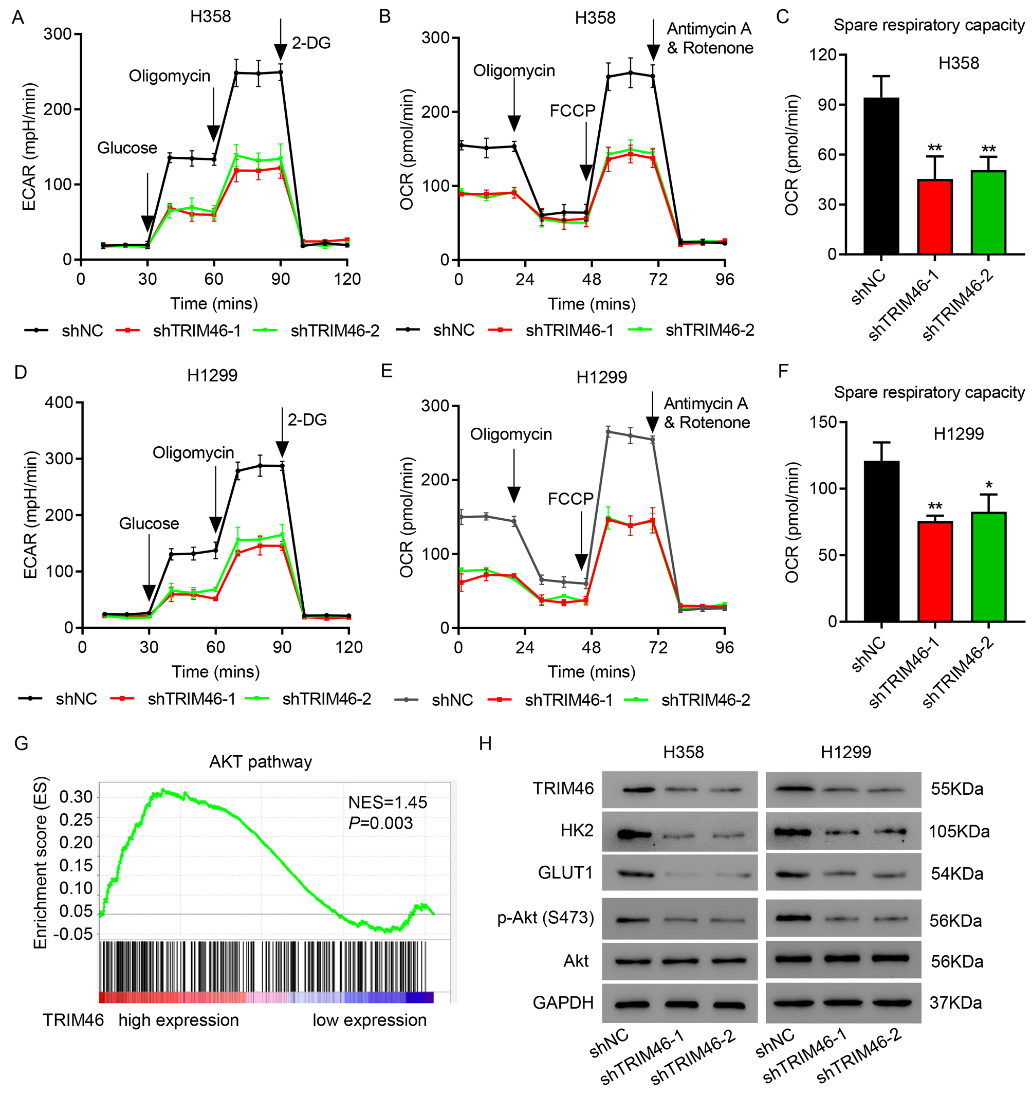


**Figure S4.** TRIM46 knockdown inhibited glycolysis in LUAD cells. (A, D) ECAR, (B, E) OCR, (C, F) spare respiratory capacity and (H) expression of TRIM46, AKT, p-AKT, HK2, and GLUT1 in both H358 and H1299 cells. (G) GSEA analysis indicated TRIM46 was correlated with AKT signaling. Results were presented as the mean ± standard error (*n* = 3). Independent experiments were repeated three times.


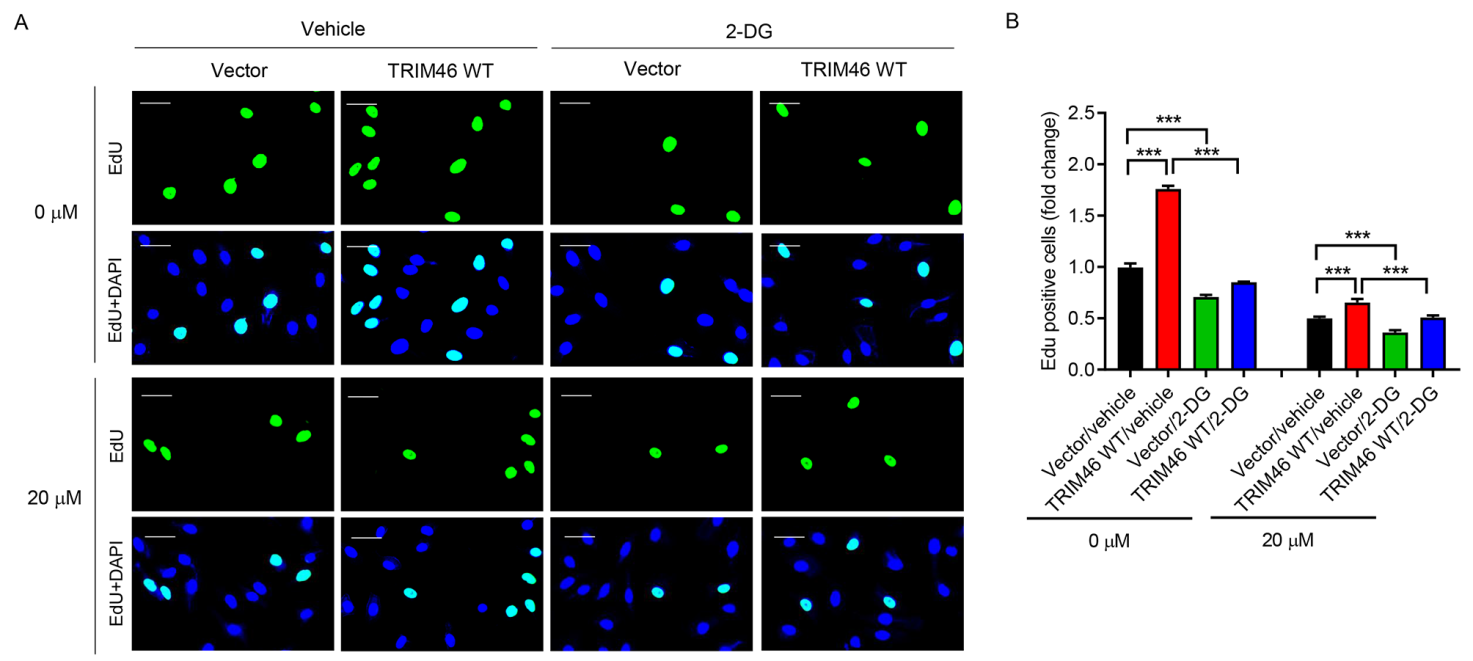


**Figure S5. TRIM46 promoted LUAD cell proliferation and DDP resistance by enhancing glycolysis.** A549 cells were transfected with WT-TRIM46 or Vector and treated by 2-DG and DDP (20 μM). (A) A549 cell proliferation measured by EdU staining (Scale bar: 50 μm). (B) Statistical analysis of EdU positive A549 cells. Results were presented as the mean ± standard error (*n* = 3). Independent experiments were repeated three times. ***P<0.001.

**
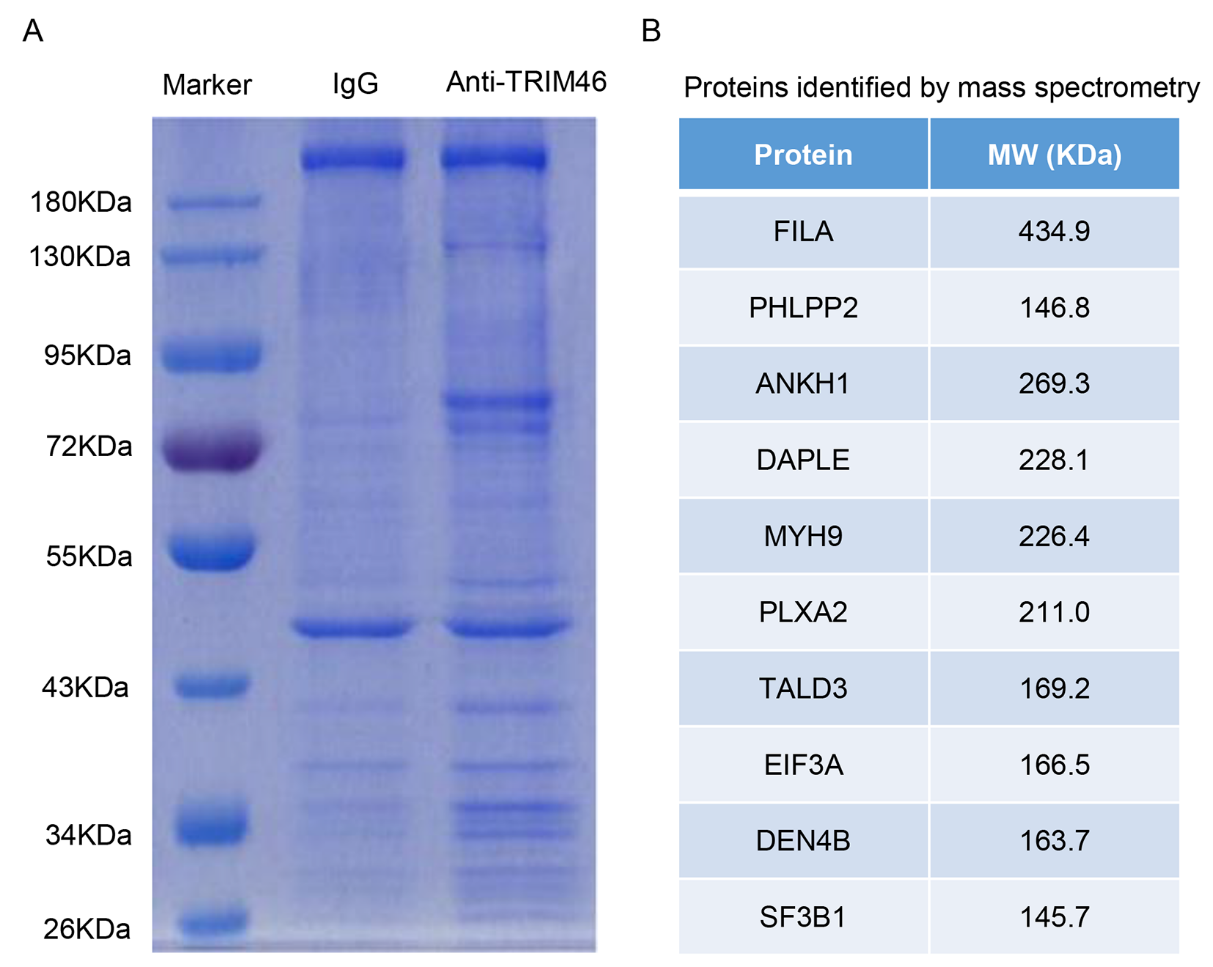
**

**Figure S6.** IP and Mass spectrometry analysis of TRIM46 binding proteins.


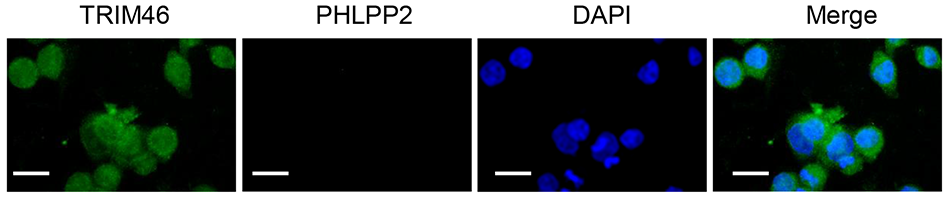


**Figure S7.** Immunofluorescence assay of TRIM46 and PHLPP2 in PHLPP2-knockout H358 cells (Scale bar: 20 μm).
